# The Microbiological Drivers of Temporally Dynamic Dimethylsulfoniopropionate Cycling Processes in Australian Coastal Shelf Waters

**DOI:** 10.3389/fmicb.2022.894026

**Published:** 2022-06-15

**Authors:** James O’Brien, Erin L. McParland, Anna R. Bramucci, Martin Ostrowski, Nachshon Siboni, Timothy Ingleton, Mark V. Brown, Naomi M. Levine, Bonnie Laverock, Katherina Petrou, Justin Seymour

**Affiliations:** ^1^Climate Change Cluster, University of Technology Sydney, Ultimo, NSW, Australia; ^2^School of Life Sciences, University of Technology Sydney, Ultimo, NSW, Australia; ^3^Department of Marine Chemistry and Geochemistry, Woods Hole Oceanographic Institution, Woods Hole, MA, United States; ^4^Water, Wetlands and Coastal Science, NSW Department of Planning, Industry and Environment, Lidcombe, NSW, Australia; ^5^School of Environmental and Life Sciences, The University of Newcastle, Callaghan, NSW, Australia; ^6^Department of Biological Sciences, University of Southern California, Los Angeles, CA, United States

**Keywords:** DMSP, DMS, DLA, phytoplankton, bacteria, qPCR, 16S rRNA gene, 18S rRNA gene

## Abstract

The organic sulfur compounds dimethylsulfoniopropionate (DMSP) and dimethyl sulfoxide (DMSO) play major roles in the marine microbial food web and have substantial climatic importance as sources and sinks of dimethyl sulfide (DMS). Seasonal shifts in the abundance and diversity of the phytoplankton and bacteria that cycle DMSP are likely to impact marine DMS (O) (P) concentrations, but the dynamic nature of these microbial interactions is still poorly resolved. Here, we examined the relationships between microbial community dynamics with DMS (O) (P) concentrations during a 2-year oceanographic time series conducted on the east Australian coast. Heterogenous temporal patterns were apparent in chlorophyll *a* (chl *a*) and DMSP concentrations, but the relationship between these parameters varied over time, suggesting the phytoplankton and bacterial community composition were affecting the net DMSP concentrations through differential DMSP production and degradation. Significant increases in DMSP were regularly measured in spring blooms dominated by predicted high DMSP-producing lineages of phytoplankton (*Heterocapsa*, *Prorocentrum*, *Alexandrium*, and *Micromonas*), while spring blooms that were dominated by predicted low DMSP-producing phytoplankton (*Thalassiosira*) demonstrated negligible increases in DMSP concentrations. During elevated DMSP concentrations, a significant increase in the relative abundance of the key copiotrophic bacterial lineage Rhodobacterales was accompanied by a three-fold increase in the gene, encoding the first step of DMSP demethylation (*dmdA*). Significant temporal shifts in DMS concentrations were measured and were significantly correlated with both fractions (0.2–2 μm and >2 μm) of microbial DMSP lyase activity. Seasonal increases of the bacterial DMSP biosynthesis gene (*dsyB*) and the bacterial DMS oxidation gene (*tmm*) occurred during the spring-summer and coincided with peaks in DMSP and DMSO concentration, respectively. These findings, along with significant positive relationships between *dsyB* gene abundance and DMSP, and *tmm* gene abundance with DMSO, reinforce the significant role planktonic bacteria play in producing DMSP and DMSO in ocean surface waters. Our results highlight the highly dynamic nature and myriad of microbial interactions that govern sulfur cycling in coastal shelf waters and further underpin the importance of microbial ecology in mediating important marine biogeochemical processes.

## Introduction

Up to 10% of the carbon fixed by marine phytoplankton is used to synthesize a single organosulfur compound called dimethylsulfoniopropionate (DMSP) (Archer et al., 2001). For DMSP-producing phytoplankton, this molecule can play a number of important physiological roles, such as an intracellular osmolyte, cryoprotectant, and antioxidant (Karsten et al., 1996; Kirst, 1996; Sunda et al., 2002). Following the release of DMSP into the surrounding water column through phytoplankton cell exudation or lysis (Bratbak et al., 1995; Stefels, 2000), this dissolved a pool of DMSP represents a major source of carbon and sulfur for heterotrophic bacteria (up to 15% of carbon and 90% of sulfur demands) (Zubkov et al., 2001, 2002). DMSP also has substantial biogeochemical importance because it is the principal precursor to the volatile gas, dimethyl sulfide (DMS). This gas is a product of phytoplankton and bacterial DMSP degradation and represents the main vehicle for the efflux of sulfur from the ocean to the atmosphere (Kettle et al., 1999; Simó, 2001), where it can subsequently be converted into cloud condensation nuclei that increase albedo (Charlson et al., 1987). However, DMS efflux and its subsequent climatic importance can be limited by transformation of the volatile gas to dimethyl sulfoxide (DMSO) through photolysis and microbial DMS oxidation (Brimblecombe and Shooter, 1986; Kiene and Bates, 1990; Lidbury et al., 2016; Thume et al., 2018). Due to the physiological, ecological, and biogeochemical importance of DMS (O) (P), an improved understanding of the temporal dynamics of the microbial community that control DMSP cycling is crucial for understanding marine ecosystem function (Kiene et al., 2000; Kiene and Linn, 2000; Simo et al., 2002; Howard et al., 2006; Levine et al., 2012; Nowinski et al., 2019b).

The production of DMSP is widespread across marine eukaryotic species, including macroalgae, brackish plants, corals, and, most notably, marine phytoplankton (Van Diggelen et al., 1986; Keller, 1989; Van Alstyne and Puglisi, 2007; McParland and Levine, 2019). While DMSP biosynthesis is performed by a large diversity of phytoplankton, there is substantial variability in the amount of DMSP produced by different phytoplankton species (Keller et al., 1989; Caruana and Malin, 2014; McParland and Levine, 2019). Generally, it is recognized that prymnesiophytes and dinoflagellates are high DMSP producers (HiDPs), while diatoms and cyanobacteria are low DMSP producers (LoDPs) (Keller, 1989; McParland and Levine, 2019). Recent evidence has reported a significant correlation between previous measurements of high and low DMSP concentrations in marine phytoplankton isolates, with the presence of two recently identified DMSP biosynthesis genes, *DSYB* and *TpMT2*, respectively (Curson et al., 2018; Kageyama et al., 2018; McParland et al., 2021). Therefore, the expression of these separate DMSP biosynthesis genes may have an important role in determining differential DMSP production in marine phytoplankton.

A majority of DMSP produced by HiDP and LoDP phytoplankton is released into the marine environment where it is available for bacterial transformations (Simo et al., 2002). Heterotrophic bacteria can transform DMSP using two different DMSP degradation pathways (Curson et al., 2011). These include the DMSP lyase pathway, encoded by the *ddd* genes, which yields DMS (Curson et al., 2011; Sun et al., 2016; Li et al., 2021), and the DMSP demethylation pathway, which is encoded by the *dmdA* gene and allows for the assimilation of DMSP-derived carbon and sulfur, but, notably, does not produce DMS (Howard et al., 2008; Reisch et al., 2008; Dupont et al., 2012). DMSP degradation through these competing cleavage and demethylation pathways is widespread in marine bacterial communities, with diverse species able to mediate one or the other, or both pathways (Simó, 2001; Reisch et al., 2011; Moran et al., 2012; Varaljay et al., 2015; Nowinski et al., 2019a). The *dmdA* gene is the most abundant DMSP degradation gene (Landa et al., 2016) and is found in dominant bacterial lineages, including the SAR11, SAR86, SAR116, and Roseobacter clades (Howard et al., 2008; Reisch et al., 2008; Dupont et al., 2012). There are also eight known non-homologous *ddd* genes (Curson et al., 2011; Sun et al., 2016; Li et al., 2021). These genes have been identified in diverse lineages of bacteria, including members of the SAR116 and Roseobacter clade (Todd et al., 2011, 2012; Choi et al., 2015), and, most recently, with the discovery of *dddK* in the SAR11 clade (Sun et al., 2021). Of these genes, *dddK* has been reported as the most dominant in pelagic open water environments (Teng et al., 2021), while the most abundant in productive coastal waters is the Roseobacter and SAR116-associated *dddP* (Nowinski et al., 2019a).

Bacterial DMS oxidation transforms DMS to DMSO and is the primary process of DMS removal in marine surface waters (Kiene and Bates, 1990). DMS oxidation is performed by bacteria that possess one of three known enzymes, a multicomponent monooxygenase (DsoABCDEF), a DMS hydrogenase (DdhABC), and trimethylamine monooxygenase (Tmm) (Horinouchi et al., 1999; McDevitt et al., 2002; Chen et al., 2011). The most abundant gene encoding bacterial DMS oxidation is *tmm* (Teng et al., 2021), which requires methylamines to convert DMS to DMSO, is estimated to be found in 20% of all bacterial cells and is notably found in the SAR11 clade and Roseobacter group (Chen et al., 2011; Lidbury et al., 2016).

Somewhat intriguingly, it has recently been shown that some non-cyanobacterial marine bacteria, including the Alphaproteobacteria and Gammaproteobacteria (McParland and Levine, 2019), also have the capacity to synthesize DMSP (Curson et al., 2017). For example, the DMSP biosynthesis genes *dsyB* and *mmtN* have been identified in DMSP-producing isolates belonging to the marine Roseobacter clade and Actinobacteria (Curson et al., 2017; Williams et al., 2019; Liu et al., 2021; Sun et al., 2021). Bacteria that possess *dsyB*, which is more abundant in marine environments, have since been reported as important DMSP producers in coastal seawater, open ocean surface seawater, coastal sediments, the deep ocean, and the surface microlayer of the East China Sea (Williams et al., 2019; Sun et al., 2020, 2021; Zheng et al., 2020; Liu et al., 2021). These recent molecular insights have provided a transformative view of the role that bacteria play in the production and cycling of DMSP, but how the occurrence and ecological dynamics of these groups change seasonally is largely unestablished.

In coastal and open ocean environments, DMSP concentrations display marked seasonal variability (Kettle et al., 1999). Highest DMSP concentrations often occur in spring and are generally attributed to phytoplankton blooms (Stefels et al., 1995; Townsend and Keller, 1996; van Duyl et al., 1998; Simó and Dachs, 2002); however, DMSP concentrations are not always coupled to phytoplankton biomass (Townsend and Keller, 1996; Vila-Costa et al., 2008). Similarly, DMS concentrations also display seasonal trends, but, unlike DMSP, levels of DMS are often greatest in the summer (Simó and Dachs, 2002). Notably, there is often no clear linear relationship between DMSP and DMS concentrations, which has been attributed to differential DMS production among phytoplankton assemblages and bacterial degradation of DMSP (Levasseur et al., 1996). The abundance of bacterial genes encoding enzymes that catalyze DMS(P) degradation (e.g., *dmdA, tmm*, and *dddP*) exhibits wide geographical distributions and is detected in tropical to polar environments (Teng et al., 2021). These genes have also been shown to significantly vary in abundance over time (Nowinski et al., 2019a), although their seasonal dynamics, relationships with environmental DMS(P) levels, and links to bacterial assemblage structure have not been examined in detail.

Oceanographic time series have monitored DMSP and DMS concentrations in diverse open-ocean and coastal sites (North Sea, Atlantic Ocean, Pacific Ocean, Indian Ocean, Southern Ocean, Baltic Sea, and Mediterranean Sea) (Dacey et al., 1998; Shenoy and Patil, 2003; Vila-Costa et al., 2008; Levine et al., 2012; Varaljay et al., 2012, 2015; Zhao et al., 2021). Although, with a recently improved understanding of the molecular mechanisms underpinning DMSP production and degradation, a combination of biogeochemical and molecular ecology approaches applied to ocean time series will deliver an even greater capacity to elucidate how the microbial community influences variability in DMS (O) (P) over time. Here, we describe a 2-year DMSP time-series study, conducted at an oceanographic station located on the eastern Australian continental shelf. Measurements of DMS (O) (P) concentrations were combined with measurements of DMS production rates (DMSP lyase assays), quantification of bacterial DMSP cycling genes, and analysis of the diversity of the phytoplankton and bacterial communities involved in DMSP cycling. By integrating this diverse suite of measurements, we aimed to identify the ecological relationships involved in DMSP cycling, with a specific focus on the genetic potential for microbes to cycle DMSP.

## Materials and Methods

### Sampling Site Description and Collection

Seawater samples were collected monthly (February 2017–January 2019) from the Australian Integrated Marine Observing System (IMOS) National Reference Station (NRS) located at Port Hacking (34° 07.06 S, 151° 13.09 E). This long-running oceanographic time-series site is situated 7 km offshore, near the city of Sydney, Australia (population, 4.3 million). Triplicate 1-L samples were collected in autoclaved and acid-washed 1-L polycarbonate bottles from 1 m below the surface, filled ensuring no headspace, and kept in the dark to avoid photo-oxidation in a cooler during transportation to the laboratory for analysis. These samples were used for characterization of dimethyl sulfide (DMS), dimethylsulfoniopropionate (DMSP), dimethyl sulfoxide (DMSO), DMSP lyase enzyme activity, and chlorophyll *a* levels. For microbial community analysis, an individual 2-L sample (*n* = 1) was collected from the same depth and immediately filtered onto a 0.22-μm polyethersulfone membrane filter (Millipore^®^ Sterivex™) using a peristaltic pump (Watson-Marlow). For quantitative PCR (qPCR) of DMSP cycling genes, a set of triplicate 2-L samples (*n* = 3) was also collected from the same depth and filtered onto a 0.22-μm polycarbonate membrane filter (Millipore^®^) using the same methods. All filters were transported on ice in a cooler during transportation before being snap-frozen in liquid nitrogen within 4 h, and then stored at −80°C until processing.

### Physicochemical Measurements and Chlorophyll *a* Content

Physicochemical data, including sea-surface temperature (°C) and salinity (PSU), dissolved oxygen (μmol L^–1^), and inorganic nutrients: nitrate/nitrites (μmol L^–1^), orthophosphate (μmol L^–1^), and silicate (μmol L^–1^), were retrieved from the IMOS curated Australian Ocean Data Network Portal^1^ (Supplementary Table 1).

Chlorophyll *a* (chl *a*) fluorescence (μg L^–^1) was measured using JGOFS protocols (Knap et al., 1996) with slight modification. Triplicate 300-ml surface water volumes were gently vacuum filtered onto a 0.7-μm glass fiber filter (GF/F, 25-mm diameter) to collect cells, which were submerged face down in a glass vial containing 3 ml of 90% acetone. The samples were vortexed and stored in the dark at −20°C for 24 h for extraction. One ml of a sample was loaded into a glass cuvette, and chl *a* concentration was measured using the chl *a* NA module in a Fluorometer (Turner Designs, Trilogy, Sunnyvale, CA, United States). A blank of 90% acetone was run to ensure background of fluorescence was at a minimum and chl *a* concentration was calculated using a standard curve (*R*^2^ = 0.99) made with chl *a* standard (Sigma-Aldrich C5753, St. Louis, MO, United States).

### Determination of Dimethylsulfoniopropionate, Dimethyl Sulfide, and Dimethyl Sulfoxide Concentrations

All dimethylated sulfur samples analyzed by gas chromatography were processed immediately after transportation from the vessel to the laboratory (within 4 h). The DMS samples were prepared by transferring 2 ml of unfiltered seawater into a 14-ml headspace vial that was capped (with a butyl rubber septum) and crimped (an aluminum crimp cap) before immediate headspace analysis. Dissolved DMSP (DMSPd) concentrations were prepared by gravity filtering no more than 3-ml seawater onto a 0.7 μm (nominal pore size) Whatman GF/F (25-mm diameter) to remove cells, while minimizing cell rupture (Kiene and Slezak, 2006). It should be noted the nominal pore size used to filter DMSPd does not exclude all bacteria and, as a result, could contain a small but likely negligible amount of bacterial particulate DMSPp (DMSPp). The first 2 ml of filtrate was collected in a 14-ml headspace vial before alkaline hydrolysis with 0.75-M NaOH. The sample was immediately capped, sealed, and left to rest for complete hydrolysis and equilibrium (at least 12 h) prior to analysis. To sample for total DMSP (DMSPt), 2 ml of unfiltered seawater was hydrolyzed with NaOH (0.75 M) before capping and sealing vials immediately, allowing sufficient time (at least 12 h) for total conversion of DMSPt to DMS and equilibrium before analysis. DMSPp was calculated as the difference between DMSPt and DMSPd.

Following DMSPt analysis, alkaline samples were uncapped and purged for 10 min with high purity nitrogen gas at a flow rate of 60 ml min^–1^ to remove any volatile sulfur compounds remaining from alkaline treatment. The samples were neutralized by adding 80 μL of 32% HCl, and DMSO was converted to DMS by adding 350 μL of 12% TiCl_3_ solution and immediately capped following previously described methods (Kiene and Gerard, 1994; Deschaseaux et al., 2014, 2019). Vials were immersed in a water bath at 50°C for 1 h and cooled to room temperature prior to purge-and-trap analysis on the GC-FPD as described below.

Analyses of sulfur compounds were performed on a gas chromatograph (GC-2010 Plus, Shimadzu, Japan), coupled with a flame photometric detector (FPD) set at 160°C with hydrogen and air flow rates at 40 and 60 ml min^–1^, respectively. A purge-and-trap methodology was used to analyze samples (Simo et al., 1993). Briefly, samples were sparged with high purity helium (He) to purge all volatile gas (including DMS) from the sample while trapping the DMS in a PTFE loop immersed in liquid nitrogen. After 4 min of cryotrapping, the loop was heated in warm water, allowing the DMS to desorb before injection into the GC. DMS was eluted onto a capillary column (30 m × 0.32 mm × 5 μm) heated to 130°C, using He as the carrier gas with a flow rate of 12 ml min^–1^ and a split ratio of five. Quantification of DMS was performed by integrating the peak area against a seven-point calibration curve of known DMS concentrations (1 pmol to 200 pmol).

### Determination of Dimethylsulfoniopropionate Lyase Enzyme Activity

The DMSP lyase activity (DLA) assay is used as a proxy for the activity of the phytoplankton and bacterial DMSP lyase pathway and provides a rate of DMS production from DMSP (Harada et al., 2004; Bell et al., 2007; Levine et al., 2012). DLA assays were performed *via* direct injection of 100 μL of headspace (column flow: 3.66 ml min^–1^) from two fractions (Levine et al., 2012): phytoplankton (>2.0 μm) and bacteria (0.22–2.0 μm) following the methods described in Harada et al. (2004). While these fractions are hereafter named phytoplankton and bacteria, it should be acknowledged that these are operational definitions only. The fractionation process does not guarantee the strict separation of both groups, whereby phytoplankton cells smaller than 2.0 μm could be collected in the bacteria fraction, and, alternatively, attached bacteria may be present in the phytoplankton fraction. Phytoplankton DLA (DLAp) assays were prepared by gentle vacuum filtration (<0.02 Pa) of 600-ml bulk seawater onto an autoclaved 2.0-μm polycarbonate filter (25-mm diameter). From the remaining filtrate, bacterial DLA assays were prepared by gentle filtration (<0.02 Pa) of 300 ml onto a 0.22-μm polycarbonate filter (25-mm diameter). All DLA samples were snap-frozen in liquid N_2_ and stored at −80°C until analysis. Prior to analysis, filters were thawed slowly on ice and then transferred facedown into a glass vial in 1 ml of a TRIS buffer (pH 8.0), capped with a rubber stopper, and vortexed for 10 s. After a 20-min incubation in a 20°C water bath, 20 μL of DMSP-HCl (Sigma-Aldrich, United States) was added to a final concentration of 5 mM, and the vial sealed and crimp capped following previously described methods (Harada et al., 2004; Sheehan and Petrou, 2020; Fernandez et al., 2021). While the addition of 5-mM DMSP is substantially higher than those observed in the environment (Galí and Simó, 2015), we have adopted the widely used methodology of Harada et al. (2004) to allow for inter-study comparison. DMSP lyase activity was then determined *via* direct injection of 100 μL of headspace (column flow: 3.66 ml min^–1^) as previously described (Harada et al., 2004; Sheehan and Petrou, 2020; Fernandez et al., 2021). All DLA assays were performed with a control (a TRIS buffer without DMSP addition) and a procedural control (a TRIS buffer with 5-mM DMSP addition). It should be noted that the procedural control of high substrate addition of DMSP (5 mM) and the alkaline TRIS buffer (pH 8.0) produced a measurable signal of DMS during the assays; these non-biological signals of DMS generation were deducted from the assay to calculate the microbial DLA for >2.0 μm and 0.22–2.0-μm fractions of seawater.

### DNA Extraction, Amplicon Sequencing, and Bioinformatic Analysis

DNA was extracted from filters using a modified application of the PowerWater^®^ DNA Isolation Kit (MO BIO Laboratories, Carlsbad, CA, United States, now Qiagen) (Appleyard et al., 2013). Bacterial and eukaryotic assemblages were characterized using 16S rRNA and 18S rRNA gene sequencing, respectively. For 16S rRNA sequencing, the V1–V3 region of the bacterial 16S rRNA gene was amplified using the 27F (AGAGTTTGATCMTGGCTCAG) (Lane, 1991) and 519R (GWATTACCGCGGCKGCTG) primer pairing (Lane et al., 1985) under the following thermocycling conditions: 95°C for 10 min; 35 cycles of 94°C for 30 s, 55°C for 10 s, and 72°C, followed by a final extension at 72°C for 10 min. The V4 region of the 18S rRNA gene was amplified using the TAReuk454FWD1 (CCAGCASCYGCGGTAATTCC) and a modified TAReuk-Rev3 (ACTTTCGTTCTTGATYRATGA) primer (Piredda et al., 2017), designed to be less discriminant against Haptophytes than the original TAReuk-Rev3 primer (Stoeck et al., 2010). Amplification was performed using the following thermocycling conditions: 98°C for 30 s; 10 cycles of 98°C for 10 s, 44°C for 30 s, and 72°C for 15 s; 20 cycles of 98°C for 10 s, 62°C for 30 s, and 72°C, followed by a final extension at 72°C for 7 min. All 16S and 18S rRNA amplicons were subsequently sequenced using the Illumina MiSeq platform at the Ramaciotti Centre for Genomics at the University of New South Wales.

Raw paired end reads for bacterial 16S and eukaryotic 18S rRNA genes were downloaded from Australian Microbiome Initiative data portal^2^ in August 2020. Low-quality reads with “N” bases were removed, and then forward and reverse primers were removed from sequences using cutadapt (Martin, 2011), and reads were truncated to eliminate low-quality terminal bases (16S truncated at R1 = 255, R2 = 250, 18S truncated at R1 = 250, R2 = 228), dereplicated, denoised, and merged using pseudo pooling. Then chimera removal was performed using dada2 removeBimeraDenovo (Callahan et al., 2016), and identical sequences of differing lengths were combined using the dada2 collapse no-mismatch step (the full pipeline available here: https://github.com/martinostrowski/marinemicrobes/tree/master/dada2). Bacterial ASVs were taxonomically classified using the SILVA v132 database, with a 50% Bayesian probability cut-off (Wang et al., 2007; Yilmaz et al., 2014), and eukaryotic ASVs were classified using the Protist Ribosomal Reference Database (PR2) (Guillou et al., 2012). A summary of all accession numbers, data availability, and number of reads per sample are available in Supplementary Table 2.

### Identifying Dimethylsulfoniopropionate-Producing Eukaryotes in 18S rRNA Gene Sequences

Photosynthetic protists were identified as 18S rRNA gene sequences assigned as Chlorophyta, Dinophyta, Cryptophyta, Haptophyta, Ochrophyta, Cercozoa, Syndiniales, and Sarcomonadea by PR2 taxonomy (Vaulot et al., 2021) and were extracted from the Eukaryotic dataset, resulting in a subset of 10,875 18S ASVs. A curated bioinformatic pipeline was used to classify these ASVs as potential DMSP producers by incorporating previous measurements of cellular DMSP production in monocultures (58) to assign their putative ability to produce DMSP based on phylogenetic inference (the full pipeline available here: https://doi.org/10.5281/zenodo.5090864). First, full-length 18S sequences of isolates with previously reported intracellular DMSP concentrations (*n* = 107) were collected from NCBI, including diverse taxa from Chlorophyta, Dinophyta, Haptophyta, Ochrophyta, Pelagophyta, Rhizaria, and Rhodophyta. The 18S rRNA gene sequences were aligned with the eukaryotic small subunit ribosomal RNA Rfam (RF01960) using Infernal (v 1.1) (Nawrocki and Eddy, 2013) in order to build a reference phylogeny with RAxML (v 8.0) (Stamatakis, 2014) using the GTRGAMMA model. A second alignment of the 10,875 unique ASVs was created with Infernal, and then pplacer (Matsen et al., 2010) was used to place ASVs onto the reference phylogeny. The ASVs that had significant sequence similarity (posterior probability of 90%, likelihood <−4,000), with an isolate previously identified to produce DMSP, were assumed to be DMSP producers. These “DMSP-producing ASVs” were further categorized as low DMSP producers (LoDP) or high DMSP producers (HiDP) based on SILVA taxonomic assignment (at the genus level), matching isolates with previously measured intracellular DMSP concentrations of less than or greater than 50-mM DMSP, respectively (McParland and Levine, 2019). While these putative assignments are a good representation of our understanding of intracellular DMSP concentrations in marine phytoplankton, it should be noted that, currently, it is impossible to predict these assignments with absolute certainty, as there are exceptions of HiDP and LoDP genera, having less than or greater than 50-mM concentrations, respectively. If a DMSP-producing ASV was not of the same genus (based on SILVA taxonomy) as a known DMSP-producing isolate, then it was defined as a likely producer with unknown DMSP production potential. A summary of how many predicted HiDP and LoDP ASVs were per sample is available in Supplementary Table 2.

### Characterization of Bacterial Dimethylsulfoniopropionate Cycling Genes

Quantitative PCR (qPCR) was used to determine the total abundance of bacterial 16S rRNA genes and genes involved in marine DMSP cycling. All qPCR analyses were performed using an epMotion 5075l automated Liquid Handling System on a Bio-Rad CFX Touch Real-Time PCR Detection System. All sample plates included a triplicate, six-point calibration curve constructed from a known amount of amplicon DNA measured by Qubit (according to the manufacturer’s instructions), followed by five successive 10-fold dilutions and negative controls of nuclease-free water. Absolute quantification of DMSP cycling genes encoding DMSP catabolism *dddP* (Levine et al., 2012) and *dmdA* (A/1 (Roseobacter), D/all (SAR11 clade), subclade (Varaljay et al., 2010), bacterial DMS oxidation (Lidbury et al., 2016), and the bacterial DMSP biosynthesis gene, *dsyB* (Williams et al., 2019) was performed using primers and annealing temperatures listed in Table 1. All assays incorporated technical triplicates of the following mixture: 2.5 μL of 2X SensiFAST SYBR Hi-ROX Master Mix, a 0.2-μL, 10-μM forward primer; a 0.2-μL, 10-μM reverse primer; 0.1-μL nuclease-free water, and 2 μL of neat DNA template. Quantification of DMSP cycling genes consisted of an initial denaturation step of 95°C for 5 min, followed by 40 cycles of 95°C for 30 s, the specified annealing temperature for each gene in Table 1 for 30 s and 72°C for 30 s. To differentiate specific amplicons from non-specific products, a dissociation melt curve was generated after each reaction.

**TABLE 1:** Primers and amplification conditions for quantitative PCR of bacterial DMSP cycling genes.

Gene	Primer	Sequence (5′-3′)	Amplicon length (bp)	Annealing temp (°C)
*dsyB*	*dsyB*-F	CATGGGSTCSAAGGCSCTKTT	246	60
	*dsyB*-R	GCAGRTARTCGCCGAAATCGTA		
*dddP*	874F	AAYGAAATWGTTGCCTTTGA	97	41
	971R	GCATDGCRTAAATCATATC		
*dmdA*(A/1)	A/1F	ATGGTGATTTGCTTCAGTTTCT	228	53
	A/1R	CCCTGCTTTGACCAACC		
*dmdA*(D/all)	D/allF	TATTGGTATAGCTATGAT	105	42
	D/allR	TAAATAAAAGGTAAATCGC		
*tmm*	tmm_RTF	CCGGCTACAAGCATTTCTTC	250	60
	Tmm_RTR	GATGTCTTCGCCCTTGTGTT		

Quantification of the bacterial 16S rRNA gene was performed using an assay adopted from Suzuki et al. (2000). Each individual PCR reaction volume was 5 μL and contained 2.5 μL of iTaq Universal probes SMX (Bio-Rad), 0.1-μL TaqMan Probe Mix, TM1389F (5′–CTTGTACACACCGCCCGTC–3′), and 0.2-μL, 10-μM concentrations of 16S rRNA gene specific primers, BACT1369F (5′–CGGTGAATACGTTCYCGG–3′) and PROK1492R (5′–GGWTACCTTGTTACGACTT–3′). Quantitative PCR was performed with the following cycling conditions: 95°C for 3 min, followed by 39 cycles of 95°C for 30 s and 56°C for 60 s. The relative abundance of bacterial DMSP-degrading genes was acquired by normalizing their copy numbers to the copy number of the bacterial 16S rRNA gene, although it should be noted that some bacterial genomes have multiple copies of the 16S rRNA gene (Cui et al., 2015).

### Statistical Analyses

To test for differences in DMS (O) (P) concentrations over time and for differences in the abundance of bacterial DMSP cycling genes, the Kruskal–Wallis (KW) tests with the Bonferroni-corrected *post hoc* tests were performed using SPSS version 17.0 (SPSS Statistics, Inc., Chicago, IL, United States). Pearson’s correlations were performed to test for significant positive and negative correlations between biogeochemical measurements [DMS(P) and DLA], molecular measurements (16S/18S rRNA gene and qPCR) and environmental parameters. When comparing interdisciplinary variables, all data were log transformed before analyses to reduce error introduced by comparing variables with different units.

## Results

### Environmental Conditions

Environmental conditions at Port Hacking exhibited clear seasonal patterns throughout the time series, from February 2017 to January 2019. Sea surface temperature peaked in the austral autumn, specifically during April 2017 (23.9°C) and March 2018 (23.0°C) (Supplementary Table 1). Salinity ranged from 35.1 to 35.8 PSU and was consistently greater than 35.5 PSU between May and September in both 2017 and 2018, with the lowest salinity measured in March 2017 (Supplementary Table 1). Nutrient levels displayed clear temporal patterns, with highest levels of both nitrate/nitrite (NO_*x*_^2–^) and phosphate (PO_3_^4–^), occurring between June and September in both years, with peak concentrations in September 2017 (3.2 μmol L^–1^ NO_*x*_^2–^ and 0.3 μmol L^–1^ PO_3_^4–^) (Supplementary Table 1). The highest concentrations of silicate (SiO_3_^2–^) occurred between March and September in both years, with peak concentrations occurring in March 2017 (1.7 μmol L^–1^) (Supplementary Table 1). Average chlorophyll *a* (chl *a*) concentrations were 1.6 ± 0.29 μg L^–1^ (average ± SE), but levels of this proxy for phytoplankton biomass displayed significant shifts over time (KW test = 58.6, df = 20, *p* < 0.01), with highest levels occurring during three phytoplankton bloom events in March 2017 (4.3 ± 0.36 μg L^–1^), October 2017 (4.4 ± 0.96 μg L^–1^), and September 2018 (4.6 ± 0.16 μg L^–1^) (Supplementary Table 1, *q* < 0.05).

### Dimethylsulfoniopropionate, Dimethyl Sulfide and Dimethyl Sulfoxide Concentrations at Port Hacking National Reference Station

**FIGURE 1:**
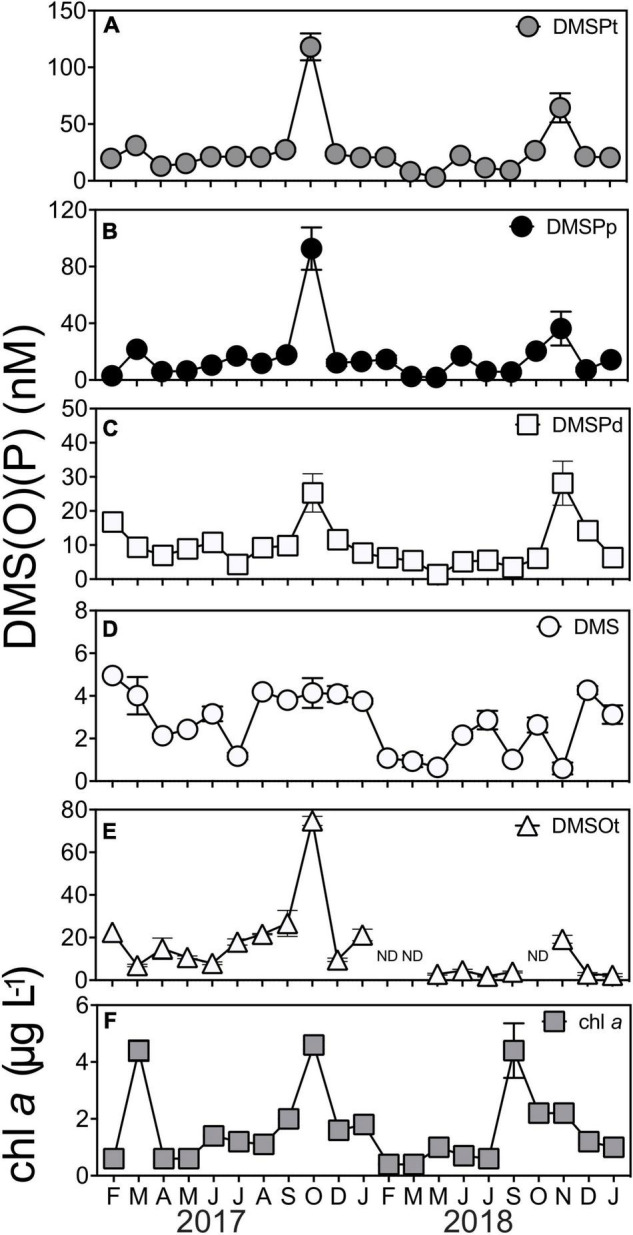
Concentrations of marine sulfur compounds **(A)** total DMSP (DMSPt) **(B)** particulate DMSP (DMSPp), **(C)** dissolved DMSP (DMSPd), **(D)** dimethyl sulfide (DMS), **(E)** total DMSO (DMSOt), and **(F)** chlorophyll a (chl *a*) at Port Hacking NRS between February 2017 and January 2019. DMS (P) data are means ± standard error (*n* = 3). ND, non-detected, indicating concentrations were below the detection limit of 1 pmol.

Clear seasonal shifts were apparent in the concentrations of total dimethylsulfoniopropionate (DMSPt, Figure 1A, KW test = 56.1, df = 20, *p* < 0.01), particulate DMSP (DMSPp, Figure 1B, KW test = 57.8 df = 20, *p* < 0.01), and dissolved DMSP (DMSPd, Figure 1C, KW test = 54.8 df = 20, *p* < 0.01). DMSPt concentrations averaged 25.7 ± 5.31 nM but increased significantly during the springtime (*q* < 0.05), whereby annual peak concentrations were measured in October 2017 (118 ± 11.8 nM) and November 2018 (64 ± 12.8 nM) (Figure 1A). Patterns in DMSPp and DMSPd concentrations were also characterized by significant seasonal increases (*q* < 0.05) in October 2017 (93 ± −14.9-nM DMSPp, 25 ± −5.6-nM DMSPd) and November 2018 (36 ± −6.0-nM DMSPp, 28 ± −6.4-nM DMSPd) (Figures 1B,C). Concentrations of the volatile sulfur compound, dimethyl sulfide (DMS), averaged 2.7 ± 0.30 nM throughout the time series (Figure 1D). Temporal patterns in DMS concentrations were not as clear as those observed for DMSP, and, notably, DMS peaks did not coincide with peaks of DMSP; instead, they occurred in the Austral summer months of February 2017 (5 ± 0.4 nM) and December 2018 (4 ± 0.2 nM) (Figure 1D). Concentrations of total dimethyl sulfoxide (DMSOt) averaged 15.1 ± 3.97 nM and demonstrated significant shifts over time (KW test = 59.2, df = 20, *p* < 0.01). Peak concentrations of DMSOt coincided with peak DMSPt in 2017, whereby DMSOt concentrations were as great as 74.8 ± 2.17 nM in October 2017 (Figure 1E).

Overall, every fraction of DMSP (DMSPt, DMSPp, and DMSPd) was significantly correlated to each other (Supplementary Table 3). A correlation between chl *a* with these fractions (DMSP, DMSPp, and DMSPd) was also detected across the duration of the time series (Supplementary Table 3), emphasizing the importance of phytoplankton biomass to DMSP cycles in the time series. This was exemplified by a high chl *a*/high DMSP event in October 2017, where peak concentrations of chl *a* and DMSPt were recorded at 4.6 ± −0.16-μg L^–1^ chl *a* and 118 ± −11.8-nM DMSPt (Figure 1F). However, despite the significant correlation found between DMSPt and chl *a* (Pearson’s *r* = 0.48, *p* < 0.05, Supplementary Table 3), not all high chl *a* events (defined as those >4-μg L^–1^ chl a) were associated with DMSP concentrations as high as October 2017. Indeed, two high chl *a*/low DMSP events were captured in the time series in March 2017 and September 2018 when phytoplankton blooms occurred (Figure 1F, as indicated by high chl *a* concentration), but, at these times, DMSPt levels were only 31 ± −0.7-nM and 27 ± −1.1-nM DMSPt, respectively (Figure 1A). Notably, during November 2018, an event characterized by low chl *a* level (2 ± 0.1 μg L^–1^) was measured to have 2-fold higher concentrations of DMSP (64 ± −12.8-nM DMSPt) than those measured during the high chl *a*/low DMSP events seen in March 2017 and September 2018 (Figures 1A,F). As well as phytoplankton biomass (chl *a*), salinity was significantly correlated with concentrations of DMSPt (Pearson’s *r* = −0.45, *p* < 0.05, Supplementary Table 3) and DMSPp (Pearson’s *r* = −0.44, *p* < 0.05, Supplementary Table 3), whereby higher levels of DMSP were associated with lower salinity measured during the time series.

No significant correlations were detected between any of the fractions of DMSP with its degradation product, DMS, or with any environmental variables (temperature, salinity, NO_*x*_^2–^, PO_3_^4–^, or SiO_3_^2–^). Additionally, there was no relationship between DMS and chl *a*, or any other environmental variable (temperature, salinity, NO_*x*_^2–^, PO_3_^4–^, or SiO_3_^2–^) (Supplementary Table 3). DMSOt concentrations were significantly positively correlated with DMSPt and DMSPd, but no significant correlation was found between DMSOt with DMS, DMSPp or any environmental variable measured throughout the 2-year time series (Supplementary Table 3).

### Temporal Dynamics of Dimethylsulfoniopropionate-Producing Eukaryotic Phytoplankton

**FIGURE 2:**
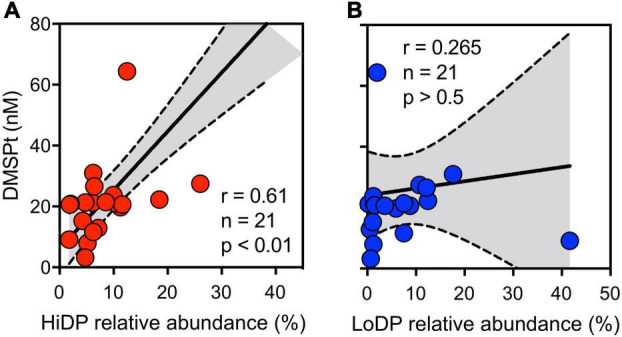
Concentration total DMSP (DMSPt), with the relative abundance of sequences putatively annotated as **(A)** high-DMSP-producing (red) and **(B)** low-DMSP-producing phytoplankton (blue) at Port Hacking NRS between February 2017 and January 2019. Pearson’s r indicates a significant correlation between variables.

Given the significant correlations between DMSPp and chl *a*, we next examined the phytoplankton community dynamics, underpinning this relationship by using 18S rRNA gene amplicon sequencing. This analysis included a consideration of the relative occurrence of predicted high DMSP-producing (HiDP) and low DMSP-producing (LoDP) phytoplankton. After characterization of putative HiDP and LoDP sequences, no significant correlation between HiDP relative abundance and chl *a* was found, although a strong significant positive correlation existed between LoDP relative abundance and chl *a* concentration (Pearson’s *r* = 0.73, *p* < 0.01, *n* = 21). Notably, there was a significant positive correlation between the relative abundance of predicted HiDPs and DMSPt concentration (Figure 2A, Pearson’s *r* = 0.61, *p* < 0.01, *n* = 21), whereas no significant correlation was evident between predicted LoDP relative abundance with DMSPt concentration (Figure 2B, Pearson’s *r* = 0.265, *p* > 0.05, *n* = 21), suggesting LoDP phytoplankton has a smaller influence on bulk DMSP concentrations than HiDP phytoplankton.

**FIGURE 3:**
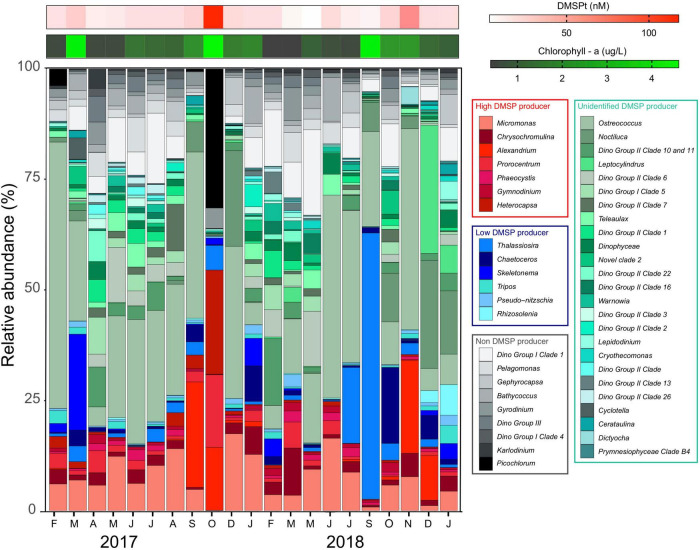
Relative abundance of eukaryotic phototrophs at Port Hacking NRS between February 2017 and January 2019. Phytoplankton taxa assigned as known high DMSP producers (red colors), known low DMSP producers (blue colors), likely producers with unknown DMSP production potential (green colors), and non-DMSP producers (gray-scale colors). Heatmaps represent concentration of total DMSP (red) and chl *a* (green).

The phytoplankton community associated with the high chl *a*/high DMSP event in October 2017 was dominated by HiDPs (48% of all 18S rRNA gene sequences) (Figure 3), with a much smaller contribution (13%) from LoDPs (Figure 3). More specifically, the phytoplankton community (the 18S phototrophic community) at this time was dominated by HiDP dinoflagellate genera, including *Heterocapsa* (24%), *Prorocentrum* (16%), *Alexandrium* (14%), and the picoeukaryote, *Picochlorum* (32%) (Figure 3).

In contrast to the patterns observed in October 2017, high chl *a*/low DMSP events in March 2017 and September 2018 were dominated by LoDPs (17 and 41% of all 18S rRNA gene sequences, respectively) (Figure 3), with a low relative abundance of HiDPs (6 and 2%, respectively) (Figure 3). The LoDP-dominated communities at these times were comprised of high relative abundances of diatoms, including *Skeletonema* (21% of 18S phototrophic sequences) in March 2017 and *Thalassiosira* (60% of 18S phototrophic sequences) in September 2018 (Figure 3).

Further evidence that suggests the abundance of predicted HiDP phytoplankton may be influencing DMSPt concentrations was demonstrated by the low chl *a*/high DMSP event in November 2018, whereby HiDPs and LoDPs were made up 12 and 2% of all 18S rRNA gene sequences, respectively (Figures 2A,B). The phytoplankton community during this event was dominated by *Ostreococcus* (46%) and a high relative abundance of the HiDP dinoflagellate, *Alexandrium* (21%) (Figure 3).

### Phytoplankton and Bacteria Dimethylsulfoniopropionate Lyase Rate Measurements

The DMSP lyase activity (DLA) assay is used as a proxy for the activity of the enzyme responsible for the cleavage of DMSP to DMS (Harada et al., 2004; Bell et al., 2007; Levine et al., 2012). Assays of operationally defined fractions of phytoplankton (DLAp, >1.2 μm) and bacteria (DLAb, 0.2–1.2 μm) revealed DLAp was significantly positively correlated with DMSPp; however, no significant correlations existed between DMSPd, DMSOt, chl *a*, and environmental variables (temperature, salinity, NO_*x*_^2–^, PO_3_^4–^, or SiO_3_^2–^) (Supplementary Table 4). Meanwhile, DLAb was not significantly correlated with DMSPp, DMSPd, DMSOt, chl *a* or environmental variables (temperature, salinity, NO_*x*_^2–^, PO_3_^4–^, or SiO_3_^2–^) (Supplementary Table 4). Notably, a significant positive correlation was detected between DLAp and DLAb with DMS (Supplementary Table 4), which demonstrates a direct link between the fractions of microbial DMSP lyase activity with its breakdown product.

**FIGURE 4:**
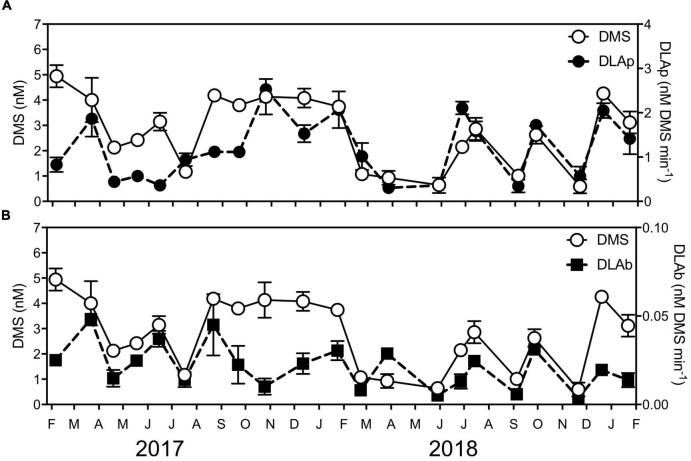
Concentrations of DMS and DMSP lyase activity (DLA) of **(A)** phytoplankton (DLAp) and **(B)** bacteria (DLAb) at Port Hacking NRS between February 2017 and January 2019. Data are means ± standard error (*n* = 3).

Although no clear seasonal trend was evident in the rate of DLAp, significant differences over time were present (KW test = 53.1, df = 20, *p* < 0.01), with highest activity (2.5 ± −0.12-nM DMS min^–1^) measured during the high chl *a*/high DMSP event in October 2017 and high chl *a*/low DMSP event in March 2017 (1.9 ± −0.40-nM DMS min^–1^) (Figure 4A). Additionally, rates of DLAb significantly shifted during the time series (KW test = 48.9, df = 20, *p* < 0.01) (Figure 4B), where, in particular, the rates measured during high chl *a*/low DMSP event in March 2017 (0.05 ± −0.001-nM DMS min^–1^) significantly exceeded those recorded in the low chl *a*/high DMSP event in November 2018 (0.004 ± −0.0004-nM DMS min^–1^) (*q* = 0.38).

### Temporal Dynamics in the Bacterial Community

**FIGURE 5:**
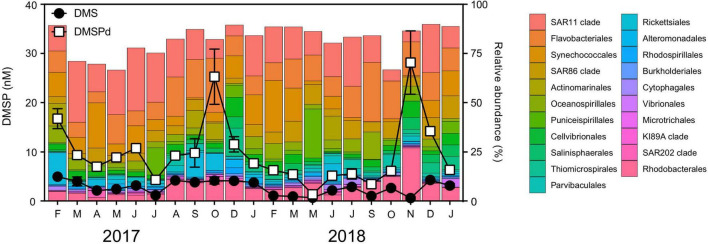
Relative abundance of the bacterial community at Port Hacking NRS between February 2017 and January 2019. Line scatter plots show nM concentrations of dissolved DMSP (DMSPd) and dimethyl sulfide (DMS) measured during the time series. DMS (P) data are means ± standard error (*n* = 3).

The dominant bacterial groups during the time series (indicated by relative abundance) were the SAR11 clade (18 ± 2.1%), Flavobacteriales (14 ± 2.1%), Synechococcales (8 ± 1.6%), Rhodobacterales (8 ± 1.2%), and the SAR86 clade (7 ± 1.2%) (Figure 5). Among the top 20 orders of bacteria, there were no significant positive relationships with any of the dimethylated sulfur compounds or chl *a* detected, with the notable exception of significant positive correlations between the relative abundance of Rhodobacterales and DMSPd (Pearson’s *r* = 0.57, *p* < 0.01, *n* = 21, Supplementary Table 5). Highest relative abundances of Rhodobacterales (∼10–27%) were evident in the austral spring to summer periods. In November 2018, during the low chl *a*/high DMSP event, Rhodobacterales dominated the bacterial community, comprising 28% of all 16S rRNA gene sequences (Figure 5). At this time, the relative abundance of Rhodobacterales was 6.6-fold, 2.8-fold, and 2.7-fold higher than during the high chl *a*/low DMSP event in March 2017, the high chl *a*/high DMSP event in October 2017, and the high chl *a*/low DMSP event in September 2018, respectively (Figure 5).

To further explore the significant increases in relative abundance of Rhodobacterales during the November 2018 DMSPd peak, we examined the dynamics of the 25 most dominant Rhodobacterales ASVs. Notably, just 5 Rhodobacterales ASVs collectively made up 21% of all 16S rRNA gene sequences in November 2018. These ASVs included HIMB11 sp. (Bc1000003) (7%), *Ascidiaceihabitans* sp. (Bc1000035, previously described as *Ascidiaceihabitans* sp. z2239) (6%), HIMB11 sp. (Bc1000116) (3%), *Amylibacter* sp. (Bc1000011, previously described as *Amylibacter* z3093) (3%), and *Planktomarina* sp. (Bc1000120, previously described as *Planktomarina* sp. z3603) (2%) (Supplementary Figure 1). Of the 5 dominant ASVs, none were significantly correlated with chl *a* throughout the time series; however, the relative abundance of *Ascidiaceihabitans* sp. (Bc1000035), HIMB11 sp. (Bc1000003), HIMB11 sp. (Bc1000116), and *Planktomarina* sp. (Bc1000120) was significantly positively correlated with DMSPd (Supplementary Figure 1). Three of these dominant ASVs [*Ascidiaceihabitans* sp. (Bc1000035), HIMB11 sp. (Bc1000116), and *Planktomarina* sp. (Bc1000120)] were greater than 10-fold more abundant in the low chl *a*/high DMSP event compared with their average abundance throughout the time series (Supplementary Figure 1).

### Temporal Dynamics of Dimethylsulfoniopropionate Degradation Genes

**FIGURE 6:**
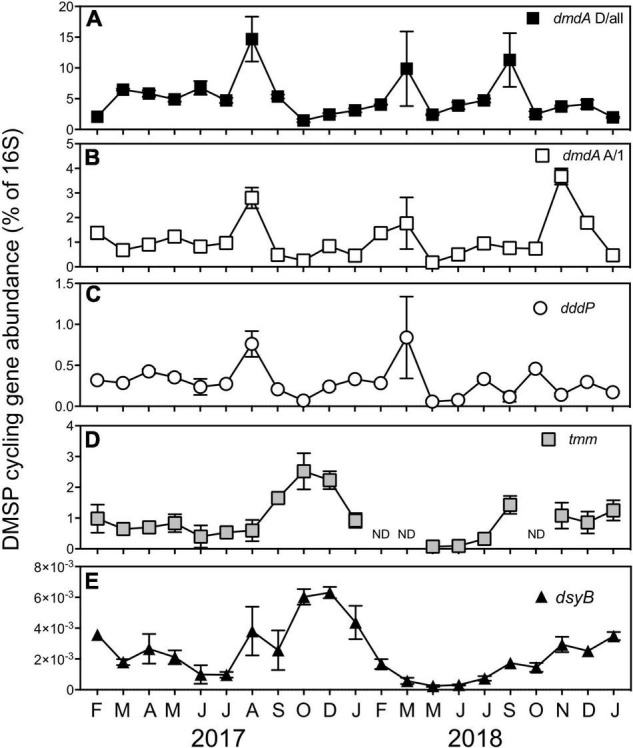
A relative proportion of DMSP cycling genes encoding the DMSP demethylation genes, **(A)** *dmdA* subclade D/all and **(B)** *dmdA* subclade A/1, **(C)** the DMSP lyase gene, *dddP*, **(D)** the bacterial DMS oxidation gene, *tmm*, and **(E)** the bacterial production gene *dsyB* at Port Hacking between February 2017 and January 2019. All data are means ± standard error (*n* = 3) and normalized to counts of the bacterial 16S rRNA gene. ND, non-detected.

Clear temporal patterns in the abundance of the genes encoding the DMSP demethylation pathway (*dmdA* subclade A/1 and D/all), the DMSP lyase pathway (*dddP*), and bacterial DMSP biosynthesis (*dsyB*) were apparent over the 21-month sampling period using quantitative polymerase chain reactions (qPCR) (Figure 6).

The DMSP demethylase genes *dmdA* subclade A/1 and *dmdA* subclade D/all are recognized as genes that belong to members of the Roseobacter clade and SAR11 clade, respectively (Levine et al., 2012; Varaljay et al., 2012). Significant temporal shifts in the abundance of both *dmdA* subclade A/1 (KW test = 49.5, df = 20, *p* < 0.01) and *dmdA* subclade D/all (KW test = 51.1, df = 20, *p* < 0.01) were observed, including concurrent significant increases (*q* < 0.05) in August 2017 and March 2018 (Figure 6). Overall, there was a significant positive correlation between *dmdA* A/1 and *dmdA* D/all (Supplementary Table 6). However, the abundance of the DMSP demethylase genes was decoupled during the high chl *a*/low DMSP event in September 2018 (Figure 6), where the abundance of *dmdA* D/all displayed a significant 2.2-fold increase (*q* < 0.05, 11 ± 4.4 copies of D/all per 16S). In contrast, the abundance of *dmdA* A/1 recorded a significant 3.3-fold increase (*q* < 0.05, 4 ± 0.3 copies of A/1 per 16S) during the low chl *a*/high DMSP event in November 2018 (Figure 6). No correlation was found between *dmdA* D/all with any dimethylated sulfur compound, DMSP lyase activity (DLAp and DLAb) or an environmental variable (temperature, salinity, chl *a*, NO_*x*_^2–^, PO_3_^4–^, or SiO_3_^2–^) (Supplementary Table 6). Despite the *dmdA* subclade D targeting SAR11 genes, no significant correlation between *dmdA* D/all and the collective relative abundance of the SAR11 order was detected, nor was the gene correlated with the abundance of other dominant bacteria (Supplementary Table 6). The abundance of the Roseobacter-associated DMSP demethylase gene (*dmdA* A/1) was significantly positively correlated with the activity of the bacterial DMSP lyase pathway (DLAb), although was not correlated with dimethylated sulfur compounds or environmental factors (Supplementary Table 6). Correlations between *dmdA* A/1 and the relative abundance of dominant orders of bacteria revealed no relationships, with exception of a significant positive correlation between *dmdA* A/1 abundance and Rhodobacterales-relative abundance (Supplementary Table 7).

With the abundance of the DMSP lyase gene, *dddP* also shifted significantly over time (KW test = 51.1, df = 20, *p* < 0.01) and was significantly positively correlated with both *dmdA* genes (Supplementary Table 6), revealing similar patterns of significantly increased abundance (*q* < 0.05) in August 2017 and March 2018 (Figure 6). The only other parameter that *dddP* displayed a significant positive correlation was DLAb, which is important as it displays a direct link between the genetic potential of *dddP* to convert DMSP to DMS, with the activity of an enzyme responsible for DMSP cleavage (Supplementary Tables 5, 6).

### Patterns in Bacterial Dimethyl Sulfide Oxidation Gene Abundance

Temporal variation was observed in the relative abundance of the gene encoding DMS oxidation *via* the trimethylamine monooxygenase enzyme, *tmm* (KW test = 31.8, df = 17, *p* = 0.01). The peak abundance of *tmm* coincided with peaks in DMSOt during the high chl a/high DMSP event in October 2017 (2.5 ± 0.59 copies of *tmm* per 16S) (Figure 6). A significant positive correlation existed between the relative abundance of *tmm* and DMSPt, DMSPd, and, notably, with DMSOt. These data give evidence to support the hypothesis that the gene *tmm*, under specific environmental conditions, may play a role in marine sulfur cycling (Supplementary Figure 2 and Supplementary Table 6). No relationship between *tmm* and environmental variables was identified during the time series (Supplementary Table 6); however, significant positive correlations were detected between *tmm* and the relative abundance of Cellvibrionales and Puniceispirillales (SAR116 clade) (Supplementary Table 7).

### Patterns in Bacterial Dimethylsulfoniopropionate Biosynthesis Gene Abundance

Seasonal patterns were observed in the abundance of the DMSP biosynthesis gene, *dsyB*. Abundances of *dsyB* showed significant increases in spring-summer months relative to winter months (*q* < 0.05), with peak abundances coinciding with the high chl *a*/high DMSP event experienced in October 2017 (0.006 ± 0.0005 copies of *dsyB* per 16S) and December 2017 (0.006 ± 0.0004 copies of *dsyB* per 16S) (Figure 6). A significant positive correlation was detected between the abundance of *dsyB* and concentrations of DMS, DMSPt, and DMSPd (Supplementary Figure 3 and Supplementary Table 6), suggesting a potential bacterial contribution to DMSP and DMS concentrations. No relationships between *dsyB* and environmental variables were found in the time-series data, although there was a significant positive correlation between *dsyB* and the relative abundance of three bacterial orders, specifically Cellvibrionales, Puniceispirillales (SAR116 clade), and Salinisphaerales (Supplementary Table 7).

## Discussion

Oceanic dimethylsulfoniopropionate (DMSP), dimethyl sulfoxide (DMSO), and dimethyl sulfide (DMS) concentrations are largely governed by ecological and metabolic interactions among marine phytoplankton and bacterial assemblages that synthesize and transform DMSP. Yet, how these interactions change over space and time and ultimately regulate the conversions among DMSP, DMSO, and DMS are poorly characterized. In this study, we coupled a suite of biological and chemical approaches to examine seasonal patterns in DMS (O) (P) cycling in coastal shelf waters in the south Pacific Ocean. Concentrations of these organosulfur compounds in our time series displayed clear seasonal trends that often share similarities with patterns observed in other coastal time series, specifically spring-time peaks of DMSP (Stefels et al., 1995; Townsend and Keller, 1996; van Duyl et al., 1998; Simó and Dachs, 2002) and summer-time peaks of DMS (Simó and Dachs, 2002). However, we revealed that, despite clear seasonal trends in DMS(P), the microbiology underpinning these dynamics is capricious.

### Spring Phytoplankton Blooms Are Sometimes, but Not Always, Accompanied by Dimethylsulfoniopropionate Pulses

**FIGURE 7:**
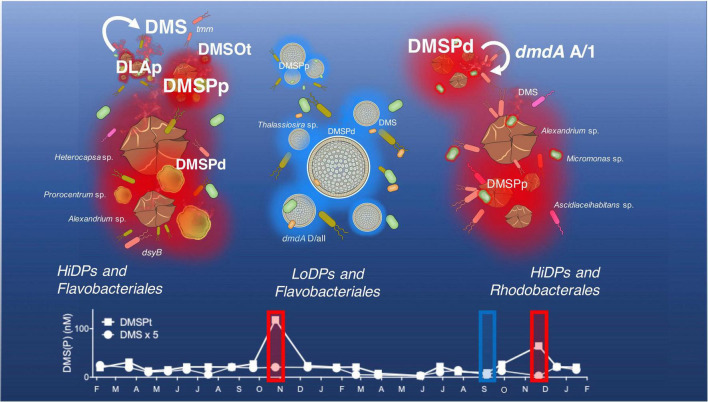
Dimethylsulfoniopropionate (DMSP)-producing phytoplankton and associated bacterial consortia influencing DMSP cycling at Port Hacking time series in October 2017 (the left panel), September 2018 (the middle panel) and November 2018 (the right panel). Concentrations of particulate DMSP (DMSPp) are driven by the composition and DMSP-producing potential of phytoplankton present. The low-DMSP-producing phenotype (LoDP) *Thalassiosira* sp. produces comparatively low-DMSPp to high-DMSP-producing phenotypes (HiDP), *Heterocapsa* sp., *Prorocentrum* sp., *Alexandrium* sp., and *Micromonas* sp. Concentrations of DMSP converted by HiDPs with active DMSP lyases enzymes (DLAp) contribute to high concentrations of the volatile sulfur emission dimethyl sulfide (DMS). High concentrations of DMS are accompanied by a high relative abundance of the bacterial DMS oxidation gene (*tmm*), which may contribute to the high dimethyl sulfoxide (DMSO) concentrations observed. The amount of DMS in the surface water at Port Hacking can be constrained by shifts in the genes encoding the DMSP demethylase pathway (*dmdA* A/1, *dmdA* D/all), including Roseobacter strains, like *Ascidiaceihabitans* sp.

Long-term observations of DMSP concentrations in eastern Australian coastal waters revealed heterogenous DMSP concentrations throughout the study. The concentrations of total DMSP (DMSPt) and particulate DMSP (DMSPp) did not display any significant correlations with temperature or any nutrient concentration (nitrate, phosphate, and silicate). No temporal relationship between DMSP with these variables is not uncommon and has previously been reported in other sulfur time series in the English channel (Archer et al., 2009), the Baltic Sea (Zhao et al., 2021), and the North Sea (Speeckaert et al., 2018). Conversely, concentrations of DMSPt and DMSPp demonstrated a significant negative correlation with salinity over the duration of the time series. This finding implies that changes in salinity play a major role in the production of DMSP by marine phytoplankton and corroborate previous research that has identified this relationship in coastal waters (Shenoy et al., 2000; Hu et al., 2005), possibly due to the hypothesized physiological role DMSP plays in marine phytoplankton as an osmolyte (Dickson and Kirst, 1986; Kirst, 1996). Shifts in DMSP observed in the time series were not just influenced by potential shifts in phytoplankton physiology but also phytoplankton biomass (chl *a*), which was significantly positively correlated with DMSPt, DMSPp, and DMSPd. Consistent with other time series (Stefels et al., 1995; Townsend and Keller, 1996; van Duyl et al., 1998; Simó and Dachs, 2002), significant annual increases of DMSP were recorded in the springtime of each year (specifically October 2017 and November 2018), which, generally, also peaked during the springtime (e.g., October 2017 and September 2018). However, our results demonstrate that elevated chl *a* levels did not always result in pulses of DMSP and that the determinants of DMSP concentrations in the study environment are more nuanced than simply the presence of phytoplankton blooms. This was clearly demonstrated by three distinctive springtime peak chl *a* and DMSP pulse events (Figure 7). The first of these events was characterized by significant peaks of both chl *a* and DMSP (a high chl *a*/high DMSP event, October 2017). The second event (September 2018) had equally high chl *a* concentrations, but much lower DMSP levels (the high chl *a*/low DMSP event), while the third event had the highest DMSP concentrations measured in 2018 (November 2018), but chl *a* levels that were half those of October 2017 (the low chl *a*/high DMSP event). These patterns indicate that springtime increases in DMSPp and DMSPd are not solely driven by phytoplankton biomass, a decoupling that might be attributed to shifts in the dominant representatives of the phytoplankton community and their DMSP-producing potential (Belviso et al., 2000; Sunda and Hardison, 2008; Archer et al., 2009).

### Pulses of Dimethylsulfoniopropionate Are Correlated With High-Dimethylsulfoniopropionate-Producing Phytoplankton Blooms

To examine the influence of successions of phytoplankton influencing DMSP dynamics in the time series, we monitored temporal shifts in the phytoplankton community, with a specific focus on DMSP production, by measuring patterns in the relative abundance of putative high-DMSP-producing phytoplankton (HiDPs) and low-DMSP-producing phytoplankton (LoDPs) (McParland and Levine, 2020). Over the duration of the time series, a significant positive relationship was measured between the relative abundance of putative HiDPs and DMSPt concentration (Figure 2A, Pearson’s *r* = 0.61, *p* < 0.01, *n* = 21). Notably, this relationship was stronger than that observed between chl *a* and DMSPt. Conversely, no relationship was found between putative LoDP relative abundance and DMSPt. The relative abundance of HiDPs and LoDPs significantly shifted over time. Springtime pulses of high DMSP (i.e., the high chl *a*/high DMSP event and the low chl *a*/high DMSP event) were always accompanied by greater relative abundance of HiDPs. Meanwhile, peaks in chl *a* that corresponded with increased LoDP-relative abundance (the high chl *a*/low DMSP event in March 2017 and the high chl *a*/low DMSP event) demonstrated negligible increases in DMSP. Our results support previous predictions based on observations from the Southern Ocean and Sargasso Sea that propose HiDP biomass dominates DMSP production, even at times when they are not the dominant population within the phytoplankton assemblage (McParland and Levine, 2019).

The HiDP phytoplankton communities corresponding with pulses of DMSP in our time series were dominated by dinoflagellates. Blooms of dinoflagellates have elsewhere been linked to high DMSP concentrations (Varaljay et al., 2015; Kiene et al., 2019; Nowinski et al., 2019a). The high chl *a*/high DMSP event was dominated by a mixed dinoflagellate bloom of *Heterocapsa*, *Prorocentrum*, and *Alexandrium* strains that were closely related to known HiDP phytoplankton isolates (Supplementary Table 8). During the low chl *a*/high DMSP event, the HiDP phytoplankton community was comprised of dominant strains with closely related HiDP isolates, including the dinoflagellate *Alexandrium* and the picoeukaryote *Micromonas* (Supplementary Table 8). Conversely, the high chl a/low DMSP event was dominated by the LoDP diatom, *Thalassiosira*. The dominant *Thalassiosira* ASV (Eb1000094) was found to be related to a known LoDP *Thalassiosira* isolate (Supplementary Table 8). Overall, our data imply that seasonal increases of *in situ* DMSP concentration are a result of increased relative abundance of predicted HiDP phytoplankton lineages and that examples of decoupling between DMSP and chl *a* are a result of predicted LoDP phytoplankton blooms. This proposed relationship between HiDP-relative abundance and DMSPt concentration is consistent with a previous study, which considered HiDP and LoDP biomass in high-performance liquid chromatography samples (HPLC) from ocean waters (McParland and Levine, 2019). However, it should be acknowledged that both approaches are predictive and that an improved representation of subdominant phytoplankton communities is required to make accurate predictions of *in situ* DMSP concentrations. Nonetheless, these results point to the importance of considering phytoplankton community composition as a major biological factor governing *in situ* DMSP concentrations, which, in turn, may influence the composition of their bacterial consortia and DMSP breakdown products.

### Succession of Key Bacterial Groups Is Correlated With Dimethylsulfoniopropionate Pulses

Temporal shifts in bacterial community composition observed during this study often corresponded with changes in chl *a* concentration and pulses of DMSP throughout the time series, providing evidence that phytoplankton-derived DMSP may play a critical role in shaping marine bacterioplankton assemblages. The strongest relationship between DMSP and any group of heterotrophic bacteria was between dissolved DMSP (DMSPd) and the Rhodobacterales. Members of the Rhodobacterales are often reported as a dominant feature of bacterial communities during blooms of DMSP-producing phytoplankton (González et al., 2000; Zubkov et al., 2002; Delmont et al., 2014). In our study, the greatest abundance of Rhodobacterales coincided with the low chl a/high DMSP *Alexandrium* and *Micromonas* bloom. During the bloom, inspection of Rhodobacterales at a higher taxonomic resolution found that five dominant amplicon sequence variants (ASVs) comprised over 20% of the entire bacterial population. Previously, we have demonstrated that two of these ASVs, *Amylibacter* sp. (Bc1000011) and *Ascidiaceihabitans* sp. (Bc1000035) display spatial and temporal relationships with the HiDP *Micromonas* at multiple time-series sites across subtropical and temperate environments (O’Brien et al., 2022). Importantly, the results of the present study demonstrate higher-than-average abundance of *Amylibacter*, *Ascidiaceihabitans*, and *Micromonas* occur during high-DMSP conditions.

Flavobacteriales were dominant members of the bacterial community coinciding with both spring high chl *a* phytoplankton blooms, although concentrations of DMSP greatly differed between the two blooms. Flavobacteria have been shown to grow while converting DMS to DMSO; however, no mechanism for this transformation has been discovered (Green et al., 2011). Instead, it is thought that the success of Flavobacteria during phytoplankton blooms is due to the breakdown and assimilation of high molecular weight carbohydrate polymers derived from phytoplankton (Kirchman, 2002; Avcı et al., 2020). In our time series, a positive correlation between the relative abundance of Flavobacteriales and chl *a* was found, but there were no significant correlations with any dimethylated sulfur compounds, indicating that the ecological links between the Flavobacteria and phytoplankton are not governed by DMS (O) (P). Overall, springtime increases in chl *a* and DMSP in the time series resulted in a shift from bacterial communities dominated by oligotrophic organisms, like the SAR11 clade (in low DMSP conditions) to copiotrophic specialists like Rhodobacterales.

### Links Between Dimethylsulfoniopropionate and Bacterial Metabolism

Within the context of marine DMS(P) cycling processes, understanding patterns in the abundance of key groups of bacteria, including the Rhodobacterales and SAR11 clade, is crucial due to their ability to transform DMSP through both the DMSP lyase (*ddd*) and DMSP demethylation (*dmdA*) pathways (Todd et al., 2009; Curson et al., 2017). The total abundance of subclades of *dmdA* belonging to Roseobacter (Subclade A/1) and the SAR11 clade (Subclade D/all) displayed heterogenous patterns in abundance during the time series. Both Roseobacter *dmdA* and SAR11 *dmdA* exhibited significant positive correlations to each other across all samples. However, the abundance of Roseobacter-associated and SAR11-associated DMSP demethylation genes decoupled in springtime 2018, whereby SAR11 *dmdA* significantly increased during the high chl a/low DMSP phytoplankton bloom, while elevated levels of Roseobacter *dmdA* were associated with the low chl a/high DMSP event. Interestingly, the springtime increase in SAR11 *dmdA* was not concomitant with above-average SAR11-relative abundance, whereas the increase in Roseobacter *dmdA* was associated with more than a three-fold increase in Rhodobacterales-relative abundance. Contrasting patterns in the abundance of these demethylation genes emphasize the differential response to DMSP displayed by the SAR11 clade and the Roseobacter group. Indeed, members of the SAR11 clade have been shown to be reliant upon reduced forms of sulfur (including DMSP) for growth (Tripp et al., 2008), although transcriptomic analysis revealed that SAR11 members do not show substantial transcriptional responses to DMSP enrichments (Vila-Costa et al., 2010), albeit this transcriptional response may be dependent upon the concentration of DMSP enrichment (Gao et al., 2020). Conversely, DMSP additions to seawater have been demonstrated to stimulate upregulation of Roseobacter-like transcripts (Vila-Costa et al., 2010). This likely explains a significant increase in the abundance of Roseobacter *dmdA*, as members of Roseobacter transform abundant DMSPd concentrations into readily assimilated reduced sulfur (Howard et al., 2008; Reisch et al., 2008; Dupont et al., 2012).

The abundance of the Roseobacter-associated DMSP lyase gene (Levine et al., 2012), *dddP*, revealed temporal heterogeneity, but no clearly discernible seasonal patterns. Overall, *dddP* was significantly positively correlated with Roseobacter demethylation genes, but, unlike *dmdA*, showed no significant increase in abundance in Spring 2018. Despite *dddP* being involved in transformations of DMSP to DMS, no significant relationship was found between the abundance of *dddP* and DMS concentrations. This is understandable, while *dddP* has been reported as the most abundant bacterial gene encoding DMSP lyase in coastal ecosystems (Nowinski et al., 2019a), it is just one of eight identified bacterial DMSP lyase genes (Todd et al., 2011, 2012; Choi et al., 2015). Notably one of these genes, *dddK*, which is found in the dominant SAR11 clade (Sun et al., 2021), can, sometimes, be just as abundant as *dddP* in coastal waters (Nowinski et al., 2019a). It is possible that the SAR11 clade may also be significant contributors of DMS; however, we did not quantify SAR11 dmdA (*dddK*) in this study. Additionally, besides bacterial degradation of DMSP, a multitude of factors control the concentration of DMS in marine surface waters, including bacterial and photo-oxidation of DMS (Toole et al., 2003; Lidbury et al., 2016), or the activity of phytoplankton and bacterial DMSP lyase enzymes (Steinke et al., 2000; Alcolombri et al., 2015).

Temporal patterns in the abundance of the bacterial DMSP degradation genes measured were significantly correlated with one another and generally exhibited increases in the late winter to springtime. A decoupling between taxon-specific genes encoding the same DMSP transformation (DMSP demethylation) highlights the importance of considering diversity of the bacterial DMSP degraders as all responses to DMSP availability are not equal (Vila-Costa et al., 2010). Moreover, significant correlations between dominant Roseobacter ASVs and DMSP degradation genes found in our data support that strain-specific responses to DMSP availability exist in marine surface waters (Nowinski et al., 2019a) and should be considered to identify key DMS(P) cycling bacteria.

### Heterogenous Biological Processes Govern Dimethyl Sulfide Production

Variable concentrations of DMS were measured over the course of the time series. While no clear seasonal trend was evident over the 2-year study, concentrations of DMS were generally greatest in the late spring to summertime. Increased DMS concentrations in summer are a global phenomenon (Bates et al., 1987; Simó and Pedrós-Alió, 1999; Williams et al., 2019) and are often thought to be a result of high-DMSP-producing phytoplankton being situated in strongly sunlit, stratified, and nutrient-depleted waters (Stefels et al., 2007; Archer et al., 2009). However, our results do not indicate a direct relationship between DMS and HiDP-associated particulate DMSP (or other fractions of DMSP). Instead, our study reveals that DMSP lyase enzyme activity by marine microbes is a greater determinant of DMS, as indicated by significant positive correlations between DMS with size fractionated microbial community samples of >2.0 μm (hereafter referred to as DLAp) and 0.22 μm–2.0 μm (hereafter referred to as DLAb) with DMS concentrations during the time series (Supplementary Table 4). Levels of both DLAp and DLAb significantly varied over time. When comparing springtime chl *a* increases and DMSP pulses observed in the time series, both DMS concentrations and DLAp rates were greater during the high chl *a*/high DMSP bloom in spring 2017 compared to the following spring. These findings highlight the significant influence that microbial DMSP lyases can have on controlling marine DMS concentrations. Despite both DLAp and DLAb being significantly positively correlated with DMS concentrations, no relationship was found between DLAp and DLAb (Pearson’s *r* = 0.17, *p* > 0.05, *n* = 21). DLAp was significantly positively correlated with DMSPp in our time series; however, previous research has suggested physical (UV-A) stress is an equally or more important determinant of DLAp rates (Levine et al., 2012). DLAb rates have been previously linked to temperature (Levine et al., 2012), although our results indicate a stronger link between DLAb rates and the abundance of the Roseobacter-associated DMSP lyase gene (*dddP*). The variable nature of DMS concentrations throughout the time series could not be explained through environmental conditions alone, and our findings demonstrate the importance of monitoring microbial DMSP lyase enzyme activity as, potentially, the most important determinant of DMS in marine surface waters.

### Links Between Dimethyl Sulfoxide and Bacterial Metabolism

Dimethylsulfoniopropionate has substantial climatic importance as a precursor compound to DMS (Charlson et al., 1987). DMSO shares this role as a DMS precursor (*via* bacterial DMSO reduction) (Bray et al., 2001), but, notably, because photochemical and microbial DMS oxidation (of DMS to DMSO) represents a major sink for DMS in the marine environment (Kiene and Bates, 1990). Bacterial DMS oxidation is the primary process for removal of DMS in marine surface waters (Lidbury et al., 2016), and the most abundant DMS oxidation pathway in the marine environment is trimethylamine monooxygenase (Tmm) (Teng et al., 2021). Tmm is encoded by the gene, *tmm*, which has been estimated to be found in 20% of all bacteria (Chen et al., 2011; Chen, 2012). Our results show that *tmm* was less abundant compared to other environments, whereby it only occurred in approximately 2.5% of bacteria (normalized to 16S rRNA genes) at Port Hacking. The relative abundance of *tmm* revealed seasonal increases, including contemporaneous peaks in *tmm* with DMSP and DMSO. This finding is significant as DMSO is a product of Tmm activity (Chen et al., 2011), although it should be acknowledged that this activity is dependent upon the presence of methylamines (Lidbury et al., 2016). Overall, these findings highlight the significance of *tmm*-carrying bacteria in contributing to seasonal surface water concentrations of DMSO, its potential role as a DMS sink, and the need for this pathway of the marine sulfur cycle to be addressed more widely, including future measurements of *in situ* methylamine concentrations, transcription, and enzyme abundance and activity.

### Bacteria as a Source of Dimethylsulfoniopropionate

Bacteria possessing the DMSP biosynthesis gene, *dsyB*, have been recognized as potentially important producers of DMSP across a multitude of marine environments (Curson et al., 2017; Williams et al., 2019; Sun et al., 2020; Zheng et al., 2020). During this study, *dsyB* levels displayed seasonal increases over spring and summer and were significantly correlated with DMSPt, DMSPd, and DMS (Supplementary Figure 2 and Supplementary Table 6). This bacterial DMSP biosynthesis gene also demonstrated greater abundances during the high chl *a*/high DMSP event compared to other significant spring blooms. The relative abundance of *dsyB* was significantly correlated with the relative abundance of a number of bacterial orders, although the reason behind these correlations is currently unclear, as *dsyB* has not yet been identified in any members of Cellvibrionales, Puniceispirillales (SAR116 clade), and Salinisphaerales. Overall, our observations indicate that bacterial DMSP production may contribute to temporal variability in ocean surface DMSP concentrations. This contribution, however, is likely to play a subsidiary role compared to HiDP production, as some HiDP dinoflagellates are known to have 17-fold greater intracellular concentrations of DMSP compared to known bacterial isolates (McParland and Levine, 2019). Nonetheless, our work and other recent studies (Williams et al., 2019; Sun et al., 2020, 2021; Zheng et al., 2020; Liu et al., 2021) confirm a need to discard a singular focus on bacteria as DMSP degraders and highlight a need to quantify the role of DMSP production by marine bacteria in marine surface waters.

### Limitations of the Study

During the sulfur time series at Port Hacking, NSW, Australia, we revealed temporal shifts in the concentration of dimethylated sulfur compounds and important factors that have the potential to govern these cycles, including microbial community composition and DMSP cycling gene abundances. It is important to note that these measurements provide robust hypotheses but do not provide absolute certainty. In order to confirm these hypotheses, it is necessary to consider the behavior of these microbial communities and, importantly, the expression of the genes in relation to *in situ* DMS (O) (P) concentrations.

## Conclusion

Identifying the ecological determinants of microbial DMSP cycling is a key to understanding their influence on climate and marine biogeochemical cycles. The present study shows that DMS (O) (P) cycling is highly complex and temporally dynamic within coastal shelf waters of the southern Pacific Ocean, with a diverse suite of processes governing surface water concentrations of DMS (O) (P) that shift in importance from one time to another. Our results emphasize that, among microbial communities, there is no “single story” behind DMSP cycling dynamics. This points to the importance of considering a wide and constantly expanding (Curson et al., 2017, 2018; McParland and Levine, 2019, 2020; McParland et al., 2021) range of biochemical processes and microbiological players involved in marine DMSP cycling.

## Data Availability Statement

The datasets presented in this study can be found in online repositories. The names of the repository/repositories and accession number(s) can be found in the article/Supplementary Material.

## Author Contributions

JO’B, JS, and KP designed the project. JO’B, NS, and TI collected the samples. MO and AB designed the sequencing data pipeline. JO’B, NS, and BL prepared qPCR pipelines used. JO’B analyzed the dataset. EM and NL developed the DMSP-producer pipeline used in the study. JO’B, AB, and MO curated the data. JS and KP acquired the funding. JO’B, AB, MO, NS, BL, MB, KP, EM, NL, and JS performed writing, review, and editing of the manuscript. All authors contributed to the article and approved the submitted version.

## Conflict of Interest

The authors declare that the research was conducted in the absence of any commercial or financial relationships that could be construed as a potential conflict of interest.

## Publisher’s Note

All claims expressed in this article are solely those of the authors and do not necessarily represent those of their affiliated organizations, or those of the publisher, the editors and the reviewers. Any product that may be evaluated in this article, or claim that may be made by its manufacturer, is not guaranteed or endorsed by the publisher.

## Footnotes

1
https://portal.aodn.org.au/

2
https://data.bioplatforms.com/organization/about/australian-microbiome

## References

[B1] Alcolombri U. Ben-Dor S. Feldmesser E. Levin Y. Tawfik D. S. Vardi A. (2015). Identification of the algal dimethyl sulfide–releasing enzyme: a missing link in the marine sulfur cycle. *Science* 348 1466–9. 10.1126/science.aab1586 26113722

[B2] Appleyard S. A. Abell G. Watson R. (2013). *Tackling Microbial Related Issues in Cultured Shellfish Via Integrated Molecular and Water Chemistry Approaches.* Hobart, Tas: CSIRO Marine and Atmospheric Research.

[B3] Archer S. D. Cummings D. G. Llewellyn C. A. Fishwick J. R. (2009). Phytoplankton taxa, irradiance and nutrient availability determine the seasonal cycle of DMSP in temperate shelf seas. *Mar. Ecol. Prog. Ser.* 394 111–24.

[B4] Archer S. D. Widdicombe C. E. Tarran G. A. Rees A. P. Burkill P. H. (2001). Production and turnover of particulate dimethylsulphoniopropionate during a coccolithophore bloom in the northern North Sea. *Aquat. Microb. Ecol.* 24 225–41.

[B5] Avcı B. Krüger K. Fuchs B. M. Teeling H. Amann R. I. (2020). Polysaccharide niche partitioning of distinct Polaribacter clades during North Sea spring algal blooms. *ISME J.* 14 1369–83. 10.1038/s41396-020-0601-y 32071394 PMC7242417

[B6] Bates T. S. Charlson R. J. Gammon R. H. (1987). Evidence for the climatic role of marine biogenic sulphur. *Nature* 329 319–21. 10.1126/science.1151109 18323445

[B7] Bell T. G. Malin G. Kim Y.-N. Steinke M. (2007). Spatial variability in DMSP-lyase activity along an Atlantic meridional transect. *Aquat. Sci.* 69 320–9.

[B8] Belviso S. Christaki U. Vidussi F. Marty J.-C. Vila M. Delgado M. (2000). Diel variations of the Dmsp-to-chlorophyll a ratio in Northwestern Mediterranean surface waters. *J. Mar. Syst.* 25 119–28.

[B9] Bratbak G. Levasseur M. Michaud S. Cantin G. Fernández E. Heimdal B. R. (1995). Viral activity in relation to Emiliania huxleyi blooms: a mechanism of DMSP release? *Mar. Ecol. Prog. Ser.* 128 133–42.

[B10] Bray R. C. Adams B. Smith A. T. Richards R. L. Lowe D. J. Bailey S. (2001). Reactions of dimethylsulfoxide reductase in the presence of dimethyl sulfide and the structure of the dimethyl sulfide-modified enzyme. *Biochemistry* 40 9810–20. 10.1021/bi010559r 11502174

[B11] Brimblecombe P. Shooter D. (1986). Photo-oxidation of dimethylsulphide in aqueous solution. *Mar. Chem.* 19 343–53.

[B12] Callahan B. J. Mcmurdie P. J. Rosen M. J. Han A. W. Johnson A. J. A. Holmes S. P. (2016). Dada2: high-resolution sample inference from Illumina amplicon data. *Nat. Methods* 13 581–3. 10.1038/nmeth.3869 27214047 PMC4927377

[B13] Caruana A. M. Malin G. (2014). The variability in DMSP content and DMSP lyase activity in marine dinoflagellates. *Prog. Oceanogr.* 120 410–24.

[B14] Charlson R. J. Lovelock J. E. Andreae M. O. Warren S. G. (1987). Oceanic phytoplankton, atmospheric sulphur, cloud albedo and climate. *Nature* 326 655–61.

[B15] Chen Y. (2012). Comparative genomics of methylated amine utilization by marine *Roseobacter clade* bacteria and development of functional gene markers (tmm, gmaS). *Environ. Microbiol.* 14 2308–22. 10.1111/j.1462-2920.2012.02765.x 22540311

[B16] Chen Y. Patel N. A. Crombie A. Scrivens J. H. Murrell J. C. (2011). Bacterial flavin-containing monooxygenase is trimethylamine monooxygenase. *Proc. Natl. Acad. Sci. U. S. A.* 108 17791–6. 10.1073/pnas.1112928108 22006322 PMC3203794

[B17] Choi D. H. Park K.-T. An S. M. Lee K. Cho J.-C. Lee J.-H. (2015). Pyrosequencing revealed SAR116 clade as dominant dddP-containing bacteria in oligotrophic NW Pacific Ocean. *PLoS One* 10:e0116271. 10.1371/journal.pone.0116271 PMC4304780 25615446

[B18] Cui Y. Suzuki S. Omori Y. Wong S.-K. Ijichi M. Kaneko R. (2015). Abundance and distribution of dimethylsulfoniopropionate degradation genes and the corresponding bacterial community structure at dimethyl sulfide hot spots in the tropical and subtropical pacific ocean. *Appl. Environ. Microbiol.* 81 4184–94. 10.1128/AEM.03873-14 25862229 PMC4524131

[B19] Curson A. R. Liu J. Martínez A. B. Green R. T. Chan Y. Carrión O. (2017). Dimethylsulfoniopropionate biosynthesis in marine bacteria and identification of the key gene in this process. *Nat. Microbiol.* 2:17009. 10.1038/nmicrobiol.2017.9 28191900

[B20] Curson A. R. Todd J. D. Sullivan M. J. Johnston A. W. (2011). Catabolism of dimethylsulphoniopropionate: microorganisms, enzymes and genes. *Nat. Rev. Microbiol.* 9 849–59. 10.1038/nrmicro2653 21986900

[B21] Curson A. R. Williams B. T. Pinchbeck B. J. Sims L. P. Martínez A. B. Rivera P. P. L. (2018). DSYB catalyses the key step of dimethylsulfoniopropionate biosynthesis in many phytoplankton. *Nat. Microbiol.* 3 430–9. 29483657 10.1038/s41564-018-0119-5

[B22] Dacey J. W. Howse F. A. Michaels A. F. Wakeham S. G. (1998). Temporal variability of dimethylsulfide and dimethylsulfoniopropionate in the Sargasso Sea. *Deep Sea Res. I Oceanogr. Res. Pap.* 45 2085–104.

[B23] Delmont T. O. Hammar K. M. Ducklow H. W. Yager P. L. Post A. F. (2014). *Phaeocystis antarctica* blooms strongly influence bacterial community structures in the Amundsen Sea polynya. *Front. Microbiol.* 5:646. 10.3389/fmicb.2014.00646 PMC4271704 25566197

[B24] Deschaseaux E. Kiene R. Jones G. B. Deseo M. A. Swan H. Oswald L. (2014). Dimethylsulphoxide (DMSO) in biological samples: a comparison of the TiCl3 and NaBH4 reduction methods using headspace analysis. *Mar. Chem.* 164 9–15.

[B25] Deschaseaux E. O’brien J. Siboni N. Petrou K. Seymour J. R. (2019). Shifts in dimethylated sulfur concentrations and microbiome composition in the red-tide causing dinoflagellate Alexandrium minutum during a simulated marine heatwave. *Biogeosciences* 16 4377–91.

[B26] Dickson D. Kirst G. (1986). The role of β-dimethylsulphoniopropionate, glycine betaine and homarine in the osmoacclimation of *Platymonas subcordiformis*. *Planta* 167 536–43. 10.1007/BF00391230 24240370

[B27] Dupont C. L. Rusch D. B. Yooseph S. Lombardo M.-J. Richter R. A. Valas R. (2012). Genomic insights to SAR86, an abundant and uncultivated marine bacterial lineage. *ISME J.* 6:1186. 10.1038/ismej.2011.189 22170421 PMC3358033

[B28] Fernandez E. Ostrowski M. Siboni N. Seymour J. R. Petrou K. (2021). Uptake of dimethylsulfoniopropionate (DMSP) by natural microbial communities of the great barrier reef (GBR), Australia. *Microorganisms* 9:1891. 10.3390/microorganisms9091891 34576786 PMC8471478

[B29] Galí M. Simó R. (2015). A meta-analysis of oceanic DMS and DMSP cycling processes: disentangling the summer paradox. *Global Biogeochem. Cycles* 29 496–515.

[B30] Gao C. Fernandez V. I. Lee K. S. Fenizia S. Pohnert G. Seymour J. R. (2020). Single-cell bacterial transcription measurements reveal the importance of dimethylsulfoniopropionate (DMSP) hotspots in ocean sulfur cycling. *Nat. Commun.* 11:1942. 10.1038/s41467-020-15693-z 32327645 PMC7181598

[B31] González J. M. Simó R. Massana R. Covert J. S. Casamayor E. O. Pedrós-Alió C. (2000). Bacterial community structure associated with a dimethylsulfoniopropionate-producing North Atlantic algal bloom. *Appl. Environ. Microbiol.* 66 4237–46. 10.1128/AEM.66.10.4237-4246.2000 11010865 PMC92291

[B32] Green D. H. Shenoy D. M. Hart M. C. Hatton A. D. (2011). Coupling of dimethylsulfide oxidation to biomass production by a marine flavobacterium. *Appl. Environ. Microbiol.* 77 3137–40. 10.1128/AEM.02675-10 21378049 PMC3126386

[B33] Guillou L. Bachar D. Audic S. Bass D. Berney C. Bittner L. (2012). The Protist Ribosomal Reference database (PR2): a catalog of unicellular eukaryote small sub-unit rRNA sequences with curated taxonomy. *Nucleic Acids Res.* 41 D597–604. 10.1093/nar/gks1160 23193267 PMC3531120

[B34] Harada H. Rouse M.-A. Sunda W. Kiene R. P. (2004). Latitudinal and vertical distributions of particle-associated dimethylsulfoniopropionate (DMSP) lyase activity in the western North Atlantic Ocean. *Can. J. Fish. Aquat. Sci.* 61 700–11.

[B35] Horinouchi M. Yoshida T. Nojiri H. Yamane H. Omori T. (1999). Polypeptide requirement of multicomponent monooxygenase DsoABCDEF for dimethyl sulfide oxidizing activity. *Biosci. Biotechnol. Biochem.* 63 1765–71. 26300166 10.1271/bbb.63.1765

[B36] Howard E. C. Henriksen J. R. Buchan A. Reisch C. R. Bürgmann H. Welsh R. (2006). Bacterial taxa that limit sulfur flux from the ocean. *Science* 314 649–52. 10.1126/science.1130657 17068264

[B37] Howard E. C. Sun S. Biers E. J. Moran M. A. (2008). Abundant and diverse bacteria involved in DMSP degradation in marine surface waters. *Environ. Microbiol.* 10 2397–410. 10.1111/j.1462-2920.2008.01665.x 18510552

[B38] Hu M. Liu L. Ma Q. Zhu T. Tian X. Dai M. (2005). Spatial–temporal distribution of dimethylsulfide in the subtropical Pearl River estuary and adjacent waters. *Continental Shelf Res.* 25 1996–2007.

[B39] Kageyama H. Tanaka Y. Shibata A. Waditee-Sirisattha R. Takabe T. (2018). Dimethylsulfoniopropionate biosynthesis in a diatom *Thalassiosira pseudonana*: identification of a gene encoding MTHB-methyltransferase. *Arch. Biochem. Biophys.* 645 100–6. 10.1016/j.abb.2018.03.019 29574051

[B40] Karsten U. Kück K. Vogt C. Kirst G. (1996). “Dimethylsulfoniopropionate production in phototrophic organisms and its physiological functions as a cryoprotectant,” in *Biological and Environmental Chemistry of DMSP and Related Sulfonium Compounds*, eds Kiene R. P. Visscher P. T. Keller M. D. Kirst G. O. (Berlin: Springer), 143–53.

[B41] Keller M. D. (1989). Dimethyl sulfide production and marine phytoplankton: the importance of species composition and cell size. *Biol. Oceanogr.* 6 375–82.

[B42] Keller M. D. Bellows W. K. Guillard R. R. (1989). *Dimethyl Sulfide Production in Marine Phytoplankton.* Washington, DC: ACS Publications.

[B43] Kettle A. Andreae M. Amouroux D. Andreae T. Bates T. Berresheim H. (1999). A global database of sea surface dimethylsulfide (DMS) measurements and a procedure to predict sea surface DMS as a function of latitude, longitude, and month. *Global Biogeochem. Cycles* 13 399–444.

[B44] Kiene R. P. Bates T. S. (1990). Biological removal of dimethyl sulphide from sea water. *Nature* 345 702–5.

[B45] Kiene R. P. Gerard G. (1994). Determination of trace levels of dimethylsulfoxide (DMSO) in seawater and rainwater. *Mar. Chem.* 47 1–12.

[B46] Kiene R. P. Linn L. J. (2000). Distribution and turnover of dissolved DMSP and its relationship with bacterial production and dimethylsulfide in the Gulf of Mexico. *Limnol. Oceanogr.* 45 849–61.

[B47] Kiene R. P. Slezak D. (2006). Low dissolved DMSP concentrations in seawater revealed by small-volume gravity filtration and dialysis sampling. *Limnol. Oceanogr. Methods* 4 80–95.

[B48] Kiene R. P. Linn L. J. Bruton J. A. (2000). New and important roles for DMSP in marine microbial communities. *J. Sea Res.* 43 209–24.

[B49] Kiene R. P. Nowinski B. Esson K. Preston C. Marin Iii R. Birch J. (2019). Unprecedented DMSP concentrations in a massive dinoflagellate bloom in Monterey Bay, CA. *Geophys. Res. Lett.* 46 12279–88.

[B50] Kirchman D. L. (2002). The ecology of *Cytophaga*–Flavobacteria in aquatic environments. *FEMS Microbiol. Ecol.* 39 91–100. 10.1111/j.1574-6941.2002.tb00910.x 19709188

[B51] Kirst G. (1996). “Osmotic adjustment in phytoplankton and macroalgae,” in *Biological and Environmental Chemistry of DMSP and Related Sulfonium Compounds*, eds Kiene R. P. Visscher P. T. Keller M. D. Kirst G. O. (Berlin: Springer), 121–9.

[B52] Knap A. Michaels A. Close A. Ducklow H. Dickson A. (1996). *Protocols for the Joint Global Ocean Flux Study (JGOFS) Core Measurements.* Paris: UNESCO.

[B53] Landa M. Blain S. Christaki U. Monchy S. Obernosterer I. (2016). Shifts in bacterial community composition associated with increased carbon cycling in a mosaic of phytoplankton blooms. *ISME J.* 10 39–50. 10.1038/ismej.2015.105 26196334 PMC4681851

[B54] Lane D. (1991). “16S/23S rRNA sequencing,” in *Nucleic Acid Techniques in Bacterial Systematics*, eds Stackenbrandt E. Goodfellow M. (Hoboken, NJ: John Wiley & Sons), 115–75.

[B55] Lane D. J. Pace B. Olsen G. J. Stahl D. A. Sogin M. L. Pace N. R. (1985). Rapid determination of 16S ribosomal RNA sequences for phylogenetic analyses. *Proc. Natl. Acad. Sci. U. S. A.* 82 6955–9. 10.1073/pnas.82.20.6955 2413450 PMC391288

[B56] Levasseur M. Michaud S. Egge J. Cantin G. Nejstgaard J. Sanders R. (1996). Production of DMSP and DMS during a mesocosm study of an Emiliania huxleyi bloom: influence of bacteria and *Calanus finmarchicus* grazing. *Mar. Biol.* 126 609–18.

[B57] Levine N. M. Varaljay V. A. Toole D. A. Dacey J. W. Doney S. C. Moran M. A. (2012). Environmental, biochemical and genetic drivers of DMSP degradation and DMS production in the Sargasso Sea. *Environ. Microbiol.* 14 1210–23. 10.1111/j.1462-2920.2012.02700.x 22324779

[B58] Li C.-Y. Wang X.-J. Chen X.-L. Sheng Q. Zhang S. Wang P. (2021). A novel ATP dependent dimethylsulfoniopropionate lyase in bacteria that releases dimethyl sulfide and acryloyl-CoA. *Elife* 10:e64045. 10.7554/eLife.64045 33970104 PMC8163506

[B59] Lidbury I. Kröber E. Zhang Z. Zhu Y. Murrell J. C. Chen Y. (2016). A mechanism for bacterial transformation of dimethylsulfide to dimethylsulfoxide: a missing link in the marine organic sulfur cycle. *Environ. Microbiol.* 18 2754–66. 10.1111/1462-2920.13354 27114231

[B60] Liu J. Zhang Y. Liu J. Zhong H. Williams B. T. Zheng Y. (2021). Bacterial dimethylsulfoniopropionate biosynthesis in the East China Sea. *Microorganisms* 9:657. 10.3390/microorganisms9030657 33810191 PMC8004995

[B61] Martin M. (2011). Cutadapt removes adapter sequences from high-throughput sequencing reads. *EMBnet J.* 17 10–2. 10.1089/cmb.2017.0096 28715235

[B62] Matsen F. A. Kodner R. B. Armbrust E. V. (2010). pplacer: linear time maximum-likelihood and Bayesian phylogenetic placement of sequences onto a fixed reference tree. *BMC Bioinformatics* 11:538. 10.1186/1471-2105-11-538 PMC3098090 21034504

[B63] McDevitt C. A. Hanson G. R. Noble C. J. Cheesman M. R. Mcewan A. G. (2002). Characterization of the redox centers in dimethyl sulfide dehydrogenase from *Rhodovulum sulfidophilum*. *Biochemistry* 41 15234–44. 10.1021/bi026221u 12484761

[B64] McParland E. L. Levine N. M. (2019). The role of differential DMSP production and community composition in predicting variability of global surface DMSP concentrations. *Limnol. Oceanogr.* 64 757–73.

[B65] McParland E. L. Levine N. M. (2020). *Evidence for Two Independent Ecological Roles of Dimethylsulfoniopropionate (DMSP).* Washington, DC: American Geophysical Union.

[B66] McParland E. L. Lee M. D. Webb E. A. Alexander H. Levine N. M. (2021). DMSP synthesis genes distinguish two types of DMSP producer phenotypes. *Environ. Microbiol.* 23 1656–69. 10.1111/1462-2920.15393 33415763

[B67] Moran M. A. Reisch C. R. Kiene R. P. Whitman W. B. (2012). Genomic insights into bacterial DMSP transformations. *Ann. Rev. Mar. Sci.* 4 523–42. 10.1146/annurev-marine-120710-100827 22457986

[B68] Nawrocki E. P. Eddy S. R. (2013). Infernal 1.1: 100-fold faster RNA homology searches. *Bioinformatics* 29 2933–5. 10.1093/bioinformatics/btt509 24008419 PMC3810854

[B69] Nowinski B. Smith C. B. Thomas C. M. Esson K. Marin R. Preston C. M. (2019b). Microbial metagenomes and metatranscriptomes during a coastal phytoplankton bloom. *Sci. Data* 6:129. 10.1038/s41597-019-0132-4 31332186 PMC6646334

[B70] Nowinski B. Motard-Côté J. Landa M. Preston C. M. Scholin C. A. Birch J. M. (2019a). Microdiversity and temporal dynamics of marine bacterial dimethylsulfoniopropionate genes. *Environ. Microbiol.* 21 1687–701. 10.1111/1462-2920.14560 30761723

[B71] O’Brien J. Mcparland E. L. Bramucci A. R. Siboni N. Ostrowski M. Kahlke T. (2022). Biogeographical and seasonal dynamics of the marine *Roseobacter* community and ecological links to DMSP-producing phytoplankton. *ISME Commun.* 2:16. 10.1038/s43705-022-00099-3 PMC9723663 37938744

[B72] Piredda R. Tomasino M. D’erchia A. Manzari C. Pesole G. Montresor M. (2017). Diversity and temporal patterns of planktonic protist assemblages at a Mediterranean long term ecological research site. *FEMS Microbiol. Ecol.* 93:fiw200. 10.1093/femsec/fiw200 27677681

[B73] Reisch C. R. Moran M. A. Whitman W. B. (2008). Dimethylsulfoniopropionate-dependent demethylase (DmdA) from *Pelagibacter ubique* and *Silicibacter pomeroyi*. *J. Bacteriol.* 190 8018–24. 10.1128/JB.00770-08 18849431 PMC2593244

[B74] Reisch C. R. Stoudemayer M. J. Varaljay V. A. Amster I. J. Moran M. A. Whitman W. B. (2011). Novel pathway for assimilation of dimethylsulphoniopropionate widespread in marine bacteria. *Nature* 473 208–11. 10.1038/nature10078 21562561

[B75] Sheehan C. E. Petrou K. (2020). Dimethylated sulfur production in batch cultures of Southern Ocean phytoplankton. *Biogeochemistry* 147 53–69.

[B76] Shenoy D. Patil J. S. (2003). Temporal variations in dimethylsulphoniopropionate and dimethyl sulphide in the Zuari estuary, Goa (India). *Mar. Environ. Res.* 56 387–402. 10.1016/S0141-1136(02)00337-9 12738221

[B77] Shenoy D. Kumar M. D. Sarma V. (2000). Controls of dimethyl sulphide in the Bay of Bengal during Bobmex-Pilot cruise 1998. *J. Earth Syst. Sci.* 109 279–283.

[B78] Simó R. (2001). Production of atmospheric sulfur by oceanic plankton: biogeochemical, ecological and evolutionary links. *Trends Ecol. Evol.* 16 287–94. 10.1016/s0169-5347(01)02152-8 11369106

[B79] Simó R. Dachs J. (2002). Global ocean emission of dimethylsulfide predicted from biogeophysical data. *Global Biogeochem. Cycles* 16:1078.

[B80] Simó R. Pedrós-Alió C. (1999). Role of vertical mixing in controlling the oceanic production of dimethyl sulphide. *Nature* 402 396–369.

[B81] Simo R. Archer S. D. Gilpin L. Stelfox-Widdicombe C. E. (2002). Coupled dynamics of dimethylsulfoniopropionate and dimethylsulfide cycling and the microbial food web in surface waters of the North Atlantic. *Limnol. Oceanogr.* 47 53–61.

[B82] Simo R. Grimalt J. O. Albaiges J. (1993). Field sampling and analysis of volatile reduced sulphur compounds in air, water and wet sediments by cryogenic trapping and gas chromatography. *J. Chromatogr. A* 655 301–7.

[B83] Speeckaert G. Borges A. V. Champenois W. Royer C. Gypens N. (2018). Annual cycle of dimethylsulfoniopropionate (DMSP) and dimethylsulfoxide (DMSO) related to phytoplankton succession in the Southern North Sea. *Sci. Total Environ.* 622 362–72. 10.1016/j.scitotenv.2017.11.359 29216471

[B84] Stamatakis A. (2014). RAxML version 8: a tool for phylogenetic analysis and post-analysis of large phylogenies. *Bioinformatics* 30 1312–3. 10.1093/bioinformatics/btu033 24451623 PMC3998144

[B85] Stefels J. (2000). Physiological aspects of the production and conversion of DMSP in marine algae and higher plants. *J. Sea Res.* 43 183–97.

[B86] Stefels J. Dijkhuizen L. Gieskes W. (1995). DMSP-lyase activity in a spring phytoplankton bloom off the Dutch coast, related to *Phaeocystis* sp. abundance. *Mar. Ecol. Prog. Ser.* 123 235–43.

[B87] Stefels J. Steinke M. Turner S. Malin G. Belviso S. (2007). Environmental constraints on the production and removal of the climatically active gas dimethylsulphide (DMS) and implications for ecosystem modelling. *Biogeochemistry* 83 245–75.

[B88] Steinke M. Malin G. Turner S. Liss P. (2000). Determinations of dimethylsulphoniopropionate (DMSP) lyase activity using headspace analysis of dimethylsulphide (DMS). *J. Sea Res.* 43 233–44.

[B89] Stoeck T. Bass D. Nebel M. Christen R. Jones M. D. Breiner H. W. (2010). Multiple marker parallel tag environmental DNA sequencing reveals a highly complex eukaryotic community in marine anoxic water. *Mol. Ecol.* 19 21–31. 10.1111/j.1365-294X.2009.04480.x 20331767

[B90] Sun H. Liu J. Tan S. Zheng Y. Wang X. Liang J. (2021). Spatiotemporal distribution of bacterial dimethylsulfoniopropionate producing and catabolic genes in the Changjiang Estuary. *Environ. Microbiol.* 23 7073–92. 10.1111/1462-2920.15813 34693622

[B91] Sun H. Zhang Y. Tan S. Zheng Y. Zhou S. Ma Q.-Y. (2020). DMSP-producing bacteria are more abundant in the surface microlayer than subsurface seawater of the East China Sea. *Microb. Ecol.* 80 350–365. 10.1007/s00248-020-01507-8 32335713

[B92] Sun J. Todd J. D. Thrash J. C. Qian Y. Qian M. C. Temperton B. (2016). The abundant marine bacterium *Pelagibacter* simultaneously catabolizes dimethylsulfoniopropionate to the gases dimethyl sulfide and methanethiol. *Nat. Microbiol.* 1:16065. 10.1038/nmicrobiol.2016.65 27573103

[B93] Sunda W. G. Hardison D. R. (2008). Contrasting seasonal patterns in dimethylsulfide, dimethylsulfoniopropionate, and chlorophyll a in a shallow North Carolina estuary and the Sargasso Sea. *Aquat. Microb. Ecol.* 53 281–94.

[B94] Sunda W. Kieber D. Kiene R. Huntsman S. (2002). An antioxidant function for DMSP and DMS in marine algae. *Nature* 418 317–20. 10.1038/nature00851 12124622

[B95] Suzuki M. T. Taylor L. T. Delong E. F. (2000). Quantitative analysis of small-subunit rRNA genes in mixed microbial populations via 5′-nuclease assays. *Appl. Environ. Microbiol.* 66 4605–14. 10.1128/AEM.66.11.4605-4614.2000 11055900 PMC92356

[B96] Teng Z.-J. Qin Q.-L. Zhang W. Li J. Fu H.-H. Wang P. (2021). Biogeographic traits of dimethyl sulfide and dimethylsulfoniopropionate cycling in polar oceans. *Microbiome* 9:207. 10.1186/s40168-021-01153-3 PMC8520302 34654476

[B97] Thume K. Gebser B. Chen L. Meyer N. Kieber D. J. Pohnert G. (2018). The metabolite dimethylsulfoxonium propionate extends the marine organosulfur cycle. *Nature* 563 412–5. 10.1038/s41586-018-0675-0 30429546

[B98] Todd J. D. Curson A. R. Kirkwood M. Sullivan M. J. Green R. T. Johnston A. W. (2011). DddQ, a novel, cupin-containing, dimethylsulfoniopropionate lyase in marine roseobacters and in uncultured marine bacteria. *Environ. Microbiol.* 13 427–38. 10.1111/j.1462-2920.2010.02348.x 20880330

[B99] Todd J. D. Kirkwood M. Newton-Payne S. Johnston A. W. (2012). DddW, a third DMSP lyase in a model Roseobacter marine bacterium, *Ruegeria pomeroyi* DSS-3. *ISME J.* 6 223–6. 10.1038/ismej.2011.79 21677693 PMC3246235

[B100] Todd J. Curson A. Dupont C. Nicholson P. Johnston A. (2009). The dddP gene, encoding a novel enzyme that converts dimethylsulfoniopropionate into dimethyl sulfide, is widespread in ocean metagenomes and marine bacteria and also occurs in some ascomycete fungi. *Environ. Microbiol.* 11 1376–85. 10.1111/j.1462-2920.2009.01864.x 19220400

[B101] Toole D. A. Kieber D. J. Kiene R. P. Siegel D. A. Nelson N. B. (2003). Photolysis and the dimethylsulfide (DMS) summer paradox in the Sargasso Sea. *Limnol. Oceanogr.* 48 1088–100.

[B102] Townsend D. W. Keller M. D. (1996). Dimethylsulfide (DMS) and dimethylsulfoniopropionate (DMSP) in relation to phytoplankton in the Gulf of Maine. *Mar. Ecol. Prog. Ser.* 137 229–41.

[B103] Tripp H. J. Kitner J. B. Schwalbach M. S. Dacey J. W. Wilhelm L. J. Giovannoni S. J. (2008). SAR11 marine bacteria require exogenous reduced sulphur for growth. *Nature* 452 741–4. 10.1038/nature06776 18337719

[B104] Van Alstyne K. L. Puglisi M. P. (2007). DMSP in marine macroalgae and macroinvertebrates: distribution, function, and ecological impacts. *Aquat. Sci.* 69 394–402.

[B105] Van Diggelen J. Rozema J. Dickson D. Broekman R. (1986). β-3-dimethylsulphoniopropionate, proline and quaternary ammonium compounds in *Spartina anglica* in relation to sodium chloride, nitrogen and sulphur. *New Phytol.* 103 573–586.

[B106] van Duyl F. C. Gieskes W. W. Kop A. J. Lewis W. E. (1998). Biological control of short-term variations in the concentration of DMSP and DMS during a *Phaeocystis* spring bloom. *J. Sea Res.* 40 221–231.

[B107] Varaljay V. A. Gifford S. M. Wilson S. T. Sharma S. Karl D. M. Moran M. A. (2012). Bacterial dimethylsulfoniopropionate degradation genes in the oligotrophic North Pacific subtropical gyre. *Appl. Environ. Microbiol.* 78 2775–82. 10.1128/AEM.07559-11 22327587 PMC3318810

[B108] Varaljay V. A. Howard E. C. Sun S. Moran M. A. (2010). Deep sequencing of a dimethylsulfoniopropionate-degrading gene (dmdA) by using PCR primer pairs designed on the basis of marine metagenomic data. *Appl. Environ. Microbiol.* 76 609–17. 10.1128/AEM.01258-09 19948858 PMC2805212

[B109] Varaljay V. A. Robidart J. Preston C. M. Gifford S. M. Durham B. P. Burns A. S. (2015). Single-taxon field measurements of bacterial gene regulation controlling DMSP fate. *ISME J.* 9 1677–1686. 25700338 10.1038/ismej.2015.23 PMC4478707

[B110] Vaulot D. Geisen S. Mahe F. Bass D. (2021). pr2-primers: an 18S rRNA primer database for protists. *bioRxiv* [Preprint]. 10.1101/2021.01.04.425170 34251760

[B111] Vila-Costa M. Kiene R. P. Simí R. (2008). Seasonal variability of the dynamics of dimethylated sulfur compounds in a coastal northwest Mediterranean site. *Limnol. Oceanogr.* 53 198–211.

[B112] Vila-Costa M. Rinta-Kanto J. M. Sun S. Sharma S. Poretsky R. Moran M. A. (2010). Transcriptomic analysis of a marine bacterial community enriched with dimethylsulfoniopropionate. *ISME J.* 4 1410–20. 10.1038/ismej.2010.62 20463763

[B113] Wang Q. Garrity G. M. Tiedje J. M. Cole J. R. (2007). Naive Bayesian classifier for rapid assignment of rRNA sequences into the new bacterial taxonomy. *Appl. Environ. Microbiol.* 73 5261–7. 10.1128/AEM.00062-07 17586664 PMC1950982

[B114] Williams B. T. Cowles K. Martínez A. B. Curson A. R. Zheng Y. Liu J. (2019). Bacteria are important dimethylsulfoniopropionate producers in coastal sediments. *Nat. Microbiol.* 4 1815–25. 10.1038/s41564-019-0527-1 31427729

[B115] Yilmaz P. Parfrey L. W. Yarza P. Gerken J. Pruesse E. Quast C. (2014). The SILVA and “all-species living tree project (LTP)” taxonomic frameworks. *Nucleic Acids Res.* 42 D643–8. 10.1093/nar/gkt1209 24293649 PMC3965112

[B116] Zhao Y. Schlundt C. Booge D. Bange H. W. (2021). A decade of dimethyl sulfide (DMS), dimethylsulfoniopropionate (DMSP) and dimethyl sulfoxide (DMSO) measurements in the southwestern Baltic Sea. *Biogeosciences* 18 2161–79.

[B117] Zheng Y. Wang J. Zhou S. Zhang Y. Liu J. Xue C.-X. (2020). Bacteria are important dimethylsulfoniopropionate producers in marine aphotic and high-pressure environments. *Nat. Commun.* 11:4658. 10.1038/s41467-020-18434-18444 32938931 PMC7494906

[B118] Zubkov M. V. Fuchs B. M. Archer S. D. Kiene R. P. Amann R. Burkill P. H. (2001). Linking the composition of bacterioplankton to rapid turnover of dissolved dimethylsulphoniopropionate in an algal bloom in the North Sea. *Environ. Microbiol.* 3 304–11. 10.1046/j.1462-2920.2001.00196.x 11422317

[B119] Zubkov M. V. Fuchs B. M. Archer S. D. Kiene R. P. Amann R. Burkill P. H. (2002). Rapid turnover of dissolved DMS and DMSP by defined bacterioplankton communities in the stratified euphotic zone of the North Sea. *Deep Sea Res. 2 Top. Stud. Oceanogr.* 49 3017–3038.

