# Supplementary material for: The Microbiological Drivers of Temporally Dynamic Dimethylsulfoniopropionate Cycling Processes in Australian Coastal Shelf Waters
Source: Front Microbiol. 2022 Jun 15;13:894026. doi: 10.3389/fmicb.2022.894026 (PMC9240709; doi:10.3389/fmicb.2022.894026)
Supplement: Supplementary file 1 [file Data_Sheet_1.pdf]

Supplementary Material

The microbiological drivers of temporally dynamic DMSP cycling processes in Australian coastal shelf waters

James O'Brien*, Erin L. McParland, Anna Bramucci, Martin Ostrowski, Nachshon Siboni, Tim Ingleton, Mark V. Brown, Naomi Levine, Katherina Petrou and Justin R. Seymour*

Supplementary Figures and Tables

Supp. Table S1 Environmental variables measured at Port hacking NRS between February 2017 to January 2019. All data was collected from the CTD cast data from IMOS (https://portal.aodn.org.au/).

**Date**	**Temp** (°C)	**Salinity** (PSU)	**NO_x** (μmol L^{-1})	**PO_3^{4-}**(μmol L^{-1})	**SiO_3^{2-}** (μmol L^{-1})
**2017**					
Feb 6	23.0	35.6	0.1	0.08	0.6
Mar 22	22.8	35.1	0.8	0.14	1.7
Apr 19	23.9	35.7	0	0.04	0.7
May 17	21.2	35.8	0.4	0.1	0.8
Jun 15	18.8	35.7	1.5	0.12	0.7
Jul 17	22.8	35.7	1	0.14	1
Aug 22	18.5	35.7	2.1	0.26	1.2
Sep 22	17.6	35.6	3.2	0.33	1.5
Oct 25	18.2	35.5	0.2	0.16	0.6
Dec 12	21.3.	35.6	0.1	0.09	0.2
**2018**					
Jan 14	21.9	35.6	0	0.11	0.6
Feb 22	19.0	35.7	0	0.09	0.9
Mar 27	23.0	35.7	0	0.04	0.3
May 29	18.8	35.7	1.7	0.24	1

Jun 27	19.4	35.7	1	0.13	0.7
Jul 14	17.1	35.7	2	0.21	0.7
Sep 5	18.3	35.7	0.4	0.13	0.9
Sep 27	19.4	35.6	1	0.18	0.9
Nov 21	19.7	35.5	0.6	0.18	0.7
Dec 19	21.8	35.5	0.1	0.05	0
**2019**					
Jan 22	18.6	35.4	0	0.06	0

Supp. Table S2 Summary table of 16S and 18S rRNA amplicon sequences, dates collected, accession numbers and available data portal where samples can be accessed. Per sample number of sequences reads, individual amplicon sequence variants (ASVs) and predicted high DMSP producing (HiDP) and low DMSP producing 18S ASVs are summarised.

Accession code	Date	Sample	Data Portal	16S reads	18S reads	16S ASVs	18S ASVs	HiDP ASVs	LoDP ASVs
SAMN26666100	6/2/17	401876	https://www.ncbi.nlm.nih.gov	67095	55389	815	476	11	10
35478	21/3/17	35478	https://data.bioplatforms.com/organization/australian-microbiome	41871	59184	1688	1121	23	36
35484	19/4/17	35484	https://data.bioplatforms.com/organization/australian-microbiome	78789	139941	671	883	24	5
35490	18/5/17	35490	https://data.bioplatforms.com/organization/australian-microbiome	50770	56696	2222	1078	19	21
35496	15/6/17	35496	https://data.bioplatforms.com/organization/australian-microbiome	46400	70757	2034	1778	43	13
35502	17/7/17	35502	https://data.bioplatforms.com/organization/australian-microbiome	50298	64267	2345	1355	31	22
139780	21/8/17	139780	https://data.bioplatforms.com/organization/australian-microbiome	78800	100621	2177	1340	42	21
SAMN26666103	22/9/17	401877	https://www.ncbi.nlm.nih.gov	37979	123793	971	725	13	11
SAMN26666102	25/10/17	401878	https://www.ncbi.nlm.nih.gov	51676	76252	744	239	8	11
139781	11/12/17	139781	https://data.bioplatforms.com/organization/australian-microbiome	56813	104836	1261	823	34	14
139782	23/1/18	139782	https://data.bioplatforms.com/organization/australian-microbiome	92084	101909	2116	1834	55	29
139783	21/2/18	139783	https://data.bioplatforms.com/organization/australian-microbiome	96757	129545	2238	1669	40	20
139784	26/3/18	139784	https://data.bioplatforms.com/organization/australian-microbiome	103395	131193	2695	2107	75	33
139785	28/5/18	139785	https://data.bioplatforms.com/organization/australian-microbiome	36090	114957	1698	1940	30	26
SAMN26666101	27/6/18	401879	https://www.ncbi.nlm.nih.gov	37979	136447	904	362	10	2
139786	24/7/18	139786	https://data.bioplatforms.com/organization/australian-microbiome	98348	102816	2609	1395	32	22

139787	5/9/18	139787	https://data.bioplatforms.com/organization/australian-microbiome	54709	136282	1386	1004	26	14
139788	27/9/18	139788	https://data.bioplatforms.com/organization/australian-microbiome	106864	105255	1981	1509	40	17
139789	20/11/18	139789	https://data.bioplatforms.com/organization/australian-microbiome	70069	118552	1357	874	36	17
139790	18/12/18	139790	https://data.bioplatforms.com/organization/australian-microbiome	113305	106228	2086	1674	37	52
139793	21/1/19	139793	https://data.bioplatforms.com/organization/australian-microbiome	29240	105728	1211	1174	36	24
					**Total**	**11594**	**7809**	**117**	**120**

Supp. Table S3 Dimethylated sulfur compounds and environmental variables measured at Port hacking NRS between February 2017 to January 2019. Bold correlations indicate significant correlation and asterisks denote ** p-value < 0.01 and * p-value <0.05.

	**DMS**	**DMSPt**	**DMSPd**	**DMSPp**	**DMSOt**	**Temp**	**Salinity**	**NO_x**	**PO_3^{4-}**	**SiO_3^{2-}**	**Chl *a***
**DMS**		0.39	0.31	0.21	0.42	0.25	-0.26	0.02	0.03	-0.13	0.16
**DMSPt**	0.39		**0.83****	**0.91****	**0.50***	0.36	**-0.45***	-0.09	0.09	0.01	**0.48***
**DMSPd**	0.31	**0.83****		**0.55***	**0.52***	0.40	-0.35	-0.22	-0.07	-0.19	**0.27***
**DMSPp**	0.21	**0.91****	**0.55***		0.38	0.21	**-0.44***	0.05	0.24	0.15	**0.59****
**DMSOt**	0.42	**0.50***	**0.52***	0.38		0.02	0.27	-0.13	0.06	0.14	0.17

Supp. Table S4 Pearson's correlation of factors influencing DMSP lyase activity in phytoplankton and bacteria. Bold correlations indicate significant correlation and asterisks denote ** p-value < 0.01 and * p-value <0.05.

	**Temp**	**Salinity**	**NO_x**	**PO_3^{4-}**	**SiO_3^{2-}**	**DMS**	**DMSPt**	**DMSPp**	**DMSPd**	**DMSOt**	**Chl *a***
**DLAp**	0.25	-0.41	-0.05	0.10	-0.14	**0.61****	**0.55***	**.582****	0.3	-0.07	0.29
**DLAb**	0.15	-0.23	0.25	0.08	0.24	**0.60****	0.04	-0.05	0.14	0.17	0.05

Supp. Table S5 Pearson's correlation of bacterial Order abundance with dimethylated sulfur compounds and chlorophyll *a* (chl *a*) measured at Port hacking NRS between February 2017 to January 2019. Bold correlations indicate significant correlation and asterisks denote ** p-value < 0.01 and * p-value <0.05.

Bacterial Order	DMS	DMSPd	DMSPt	DMSPp	DMSOt	Chl *a*
Actinomarinales	-0.15	-0.16	-0.28	-0.30	-0.32	0.2
Alteromonadales	0.09	-0.03	-0.13	-0.15	0.30	-0.14
Burkholderiales	-0.09	-0.07	0.03	0.06	-0.05	0.28
Cellvibrionales	-0.03	0.39	0.21	0.13	0.11	0.05
Cytophagales	0.04	-0.20	-0.25	-0.25	-0.21	-0.4
Flavobacteriales	-0.03	0.25	0.41	0.43	-0.03	**0.58****
KI89A_clade	-0.19	0.03	-0.14	-0.18	0.27	-0.16
Microtrichales	-0.06	-0.19	0.02	0.09	-0.16	-0.16
Oceanospirillales	-0.42	-0.32	-0.22	-0.16	-0.03	-0.19
Parvibaculales	-0.23	-0.16	-0.22	-0.22	-0.21	-0.42
Puniceispirillales	-0.03	0.33	0.14	0.07	0.44	0.10
Rhodobacterales	-0.20	**0.57****	0.41	0.32	0.15	0.27
Rhodospirillales	0.24	0.09	0.11	0.11	0.46	0.10
Rickettsiales	0.43	0.29	0.26	0.22	0.25	0.10
Salinisphaerales	0.25	0.08	-0.04	-0.08	0.13	-0.10
SAR11_clade	-0.06	-0.33	-0.38	-0.36	-0.36	-0.10
SAR202 clade	-0.15	-0.15	-0.18	-0.18	-0.01	0.10
SAR86 clade	-0.37	-0.41	-.437*	-0.41	-0.48*	-0.46*
Synechococcales	0.02	-0.14	-0.21	-0.21	0.19	-0.42
Thiomicrospirales	0.06	-0.18	-0.12	-0.08	-0.36	0.01
Vibrionales	0.09	-0.04	-0.03	-0.02	-0.16	-0.18

Supp. Table S6 Pearson's correlation of dimethylated sulfur compounds with bacterial DMSP cycling genes, bacterial DMSP lyase activity (DLAb) and environmental parameters measured at Port hacking NRS between February 2017 to January 2019. Bold correlations indicate significant correlation and asterisks denote ** p-value < 0.01 and * p-value <0.05.

	**D/all**	**A/1**	***dddP***	***tmm***	***dsyB***	**Temp**	**Salinity**	**NO_x**	**PO_3^{4-}**	**SiO_3^{2-}**
***dmdA* D/all**		**0.48***	**0.73****	-0.16	-0.24	0.09	0.12	0.27	0.16	0.33
***dmdA* A/1**	**0.48***		**0.47***	-0.01	-0.06	0.08	0.28	0.08	-0	0.09
dddP	**0.73****	**0.47***		-0.14	-0.05	0.12	0.17	-0	-0.1	-0.01
tmm	-0.16	-0.01	-0.14		**0.83****	0.14	-0.26	-0.42	-0.11	-0.26
dsyB	-0.24	-0.06	-0.05	**0.83****		-0.26	-0.2	-0.3	-0.1	-0.3
**DMS**	-0.1	0.1	0.1	0.44	**0.6****	0.25	-0.26	0.02	0.03	-0.13
**DMSPt**	-0.3	-0.3	-0.3	**0.57***	**0.54***	0.36	**-0.45***	-0.09	0.09	0.01
**DMSPd**	-0.3	-0.2	-0.2	**0.57***	**0.58***	0.40	-0.35	-0.22	-0.07	-0.19
**DMSPp**	-0.3	-0.3	-0.3	0.47*	0.43	0.21	**-0.44***	0.05	0.24	0.15
**DMSOt**	-0.06	0.19	0.24	**0.57***	**0.70****	0.02	0.27	-0.13	0.06	0.14
**DLAb**	0.39	**0.48***	**0.6****	-0.14	0.04	0.1	-0.3	0.24	0.09	0.3

Supp. Table S7 Pearson's correlation of bacterial Order abundance with bacterial DMSP-cycling gene abundance measured at Port hacking NRS between February 2017 to January 2019. Bold correlations indicate significant correlation and asterisks denote ** p-value < 0.01 and * p-value <0.05.

Bacterial Order	*dmdA* D/all	*dmdA* A/1	*dddP*	*tmm*	*dsyB*
Actinomarinales	0.36	0.08	-0.05	-0.29	-0.24
Alteromonadales	-0.28	-0.12	-0.11	0.14	0.42
Burkholderiales	-0.31	-0.13	-0.09	0.31	0.21
Cellvibrionales	-0.21	0.29	0.04	**0.60****	**0.43***
Cytophagales	0.04	-0.01	0.06	-0.63**	-0.42
Flavobacteriales	0.25	0.08	0.17	0.43	0.19
KI89A_clade	-0.08	0.27	0.19	0.08	0.28
Microtrichales	0.27	-0.06	0.20	-0.56*	-0.32
Oceanospirillales	-0.27	-0.22	-0.11	-0.26	-0.23
Parvibaculales	0.30	0.40	0.26	-0.64**	-0.38
Puniceispirillales	-0.01	0.37	0.15	**0.57***	**0.52***
Rhodobacterales	-0.04	**0.58****	0.06	0.43	0.26
Rhodospirillales	-0.18	-0.11	-0.09	0.22	0.40
Rickettsiales	-0.26	-0.30	-0.17	0.09	0.17
Salinisphaerales	-0.24	0.02	-0.03	0.42	**0.57****
SAR11_clade	0.34	-0.04	0.04	-0.36	-0.42
SAR202 clade	-0.11	-0.26	-0.18	-0.30	0.14
SAR86 clade	-0.01	-0.03	0.16	-0.31	-0.43
Synechococcales	-0.04	-0.11	0.10	-0.05	0.10
Thiomicrospirales	0.15	0.14	0.14	-0.24	-0.22
Vibrionales	-0.23	-0.17	-0.08	0.30	0.25

Supp. Table S8 BLAST output between DMSP-producing strains and known DMSP-producing isolates. Intracellular DMSP concentrations are collected from McParland and Levine (2019).

Strain	Isolate	Percent identity (%)	Query cover (%)	Intracellular DMSP (mM)
***Heterocapsa* (Eb1000123)**	*Heterocapsa rotunda*	95	100	452
***Prorocentrum* (Eb1000458)**	*Prorocentrum minimum*	**94**	**100**	**1082**
***Alexandrium* (Eb1000681)**	*Alexandrium minitum*	**99**	**100**	**200**
***Thalassiosira* (Eb1000094)**	*Thalassiosira oceanica*	**94**	**100**	**2.8**

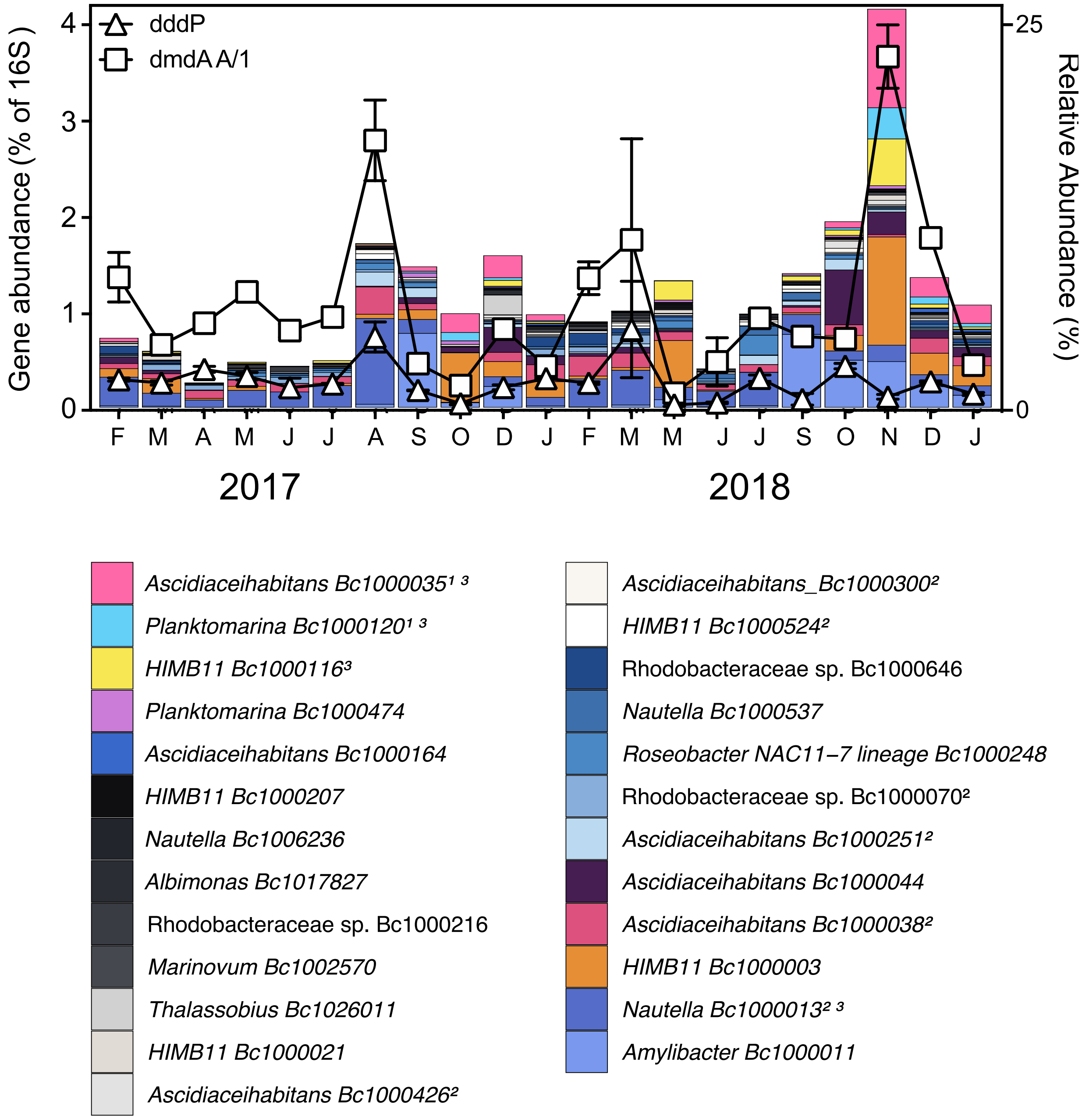

Supp. Fig. S1 Top 25 most abundant Rhodobacterales ASVs in the Port Hacking time-series (right y – axis). Symbols represent the abundance of Roseobacter-associated DMSP degradation genes *dddP* (triangle) and *dmdA* subclade A/1 (square) (left y – axis). Annotations [1,2,3] denote a significant positive Pearson's r with DMSPd. *dddP* and *dmdA* A/1 respectively.

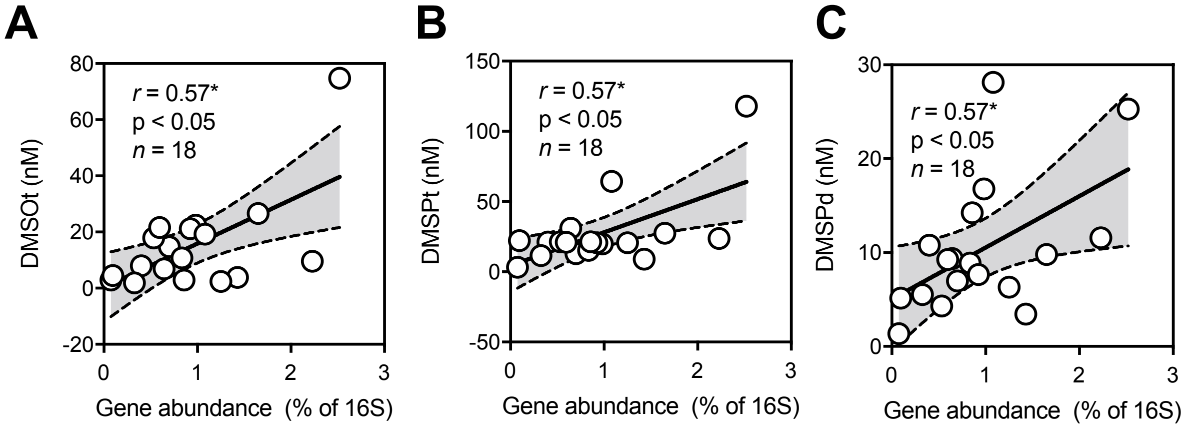

Supp. Fig. S2 Pearson's correlations between the relative abundance of the bacterial DMS oxidation gene, *tmm* with **(A)** total dimethyl sulfoxide (DMSOt), **(B)** total dimethylsulfoniopropionate (DMSPt) and **(C)** dissolved DMSP (DMSPd).

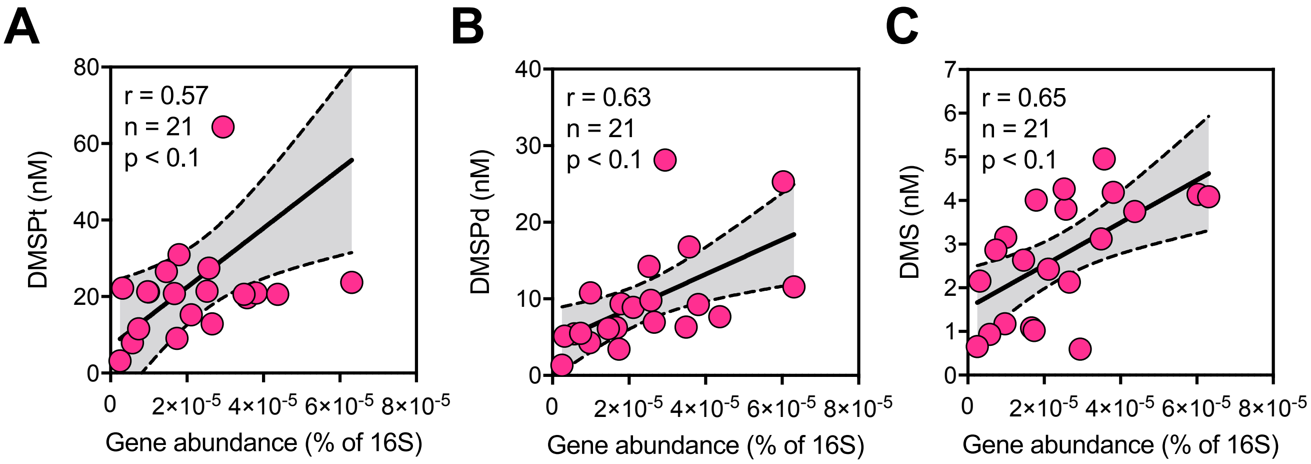

Supp. Fig. S3 Pearson's correlations between the relative abundance of the bacterial DMSP biosynthesis gene, *dsyB* with **(A)** total dimethylsulfoniopropionate (DMSPt), **(B)** dissolved DMSP (DMSPd) and **(C)** dimethylsulfide (DMS).
